# Switch from Stress Response to Homeobox Transcription Factors in Adipose Tissue After Profound Fat Loss

**DOI:** 10.1371/journal.pone.0011033

**Published:** 2010-06-09

**Authors:** Simon N. Dankel, Dag J. Fadnes, Anne-Kristin Stavrum, Christine Stansberg, Rita Holdhus, Tuyen Hoang, Vivian L. Veum, Bjørn Jostein Christensen, Villy Våge, Jørn V. Sagen, Vidar M. Steen, Gunnar Mellgren

**Affiliations:** 1 Institute of Medicine, University of Bergen, Bergen, Norway; 2 Hormone Laboratory, Haukeland University Hospital, Bergen, Norway; 3 Department of Medicine, Førde Central Hospital, Førde, Norway; 4 Department of Heart Disease, Haukeland University Hospital, Bergen, Norway; 5 Center for Medical Genetics and Molecular Medicine, Haukeland University Hospital, Bergen, Norway; 6 Department of Clinical Medicine, University of Bergen, Bergen, Norway; 7 Department of Surgery, Haukeland University Hospital, Bergen, Norway; 8 Department of Surgical Sciences, University of Bergen, Bergen, Norway; 9 Department of Surgery, Førde Central Hospital, Førde, Norway; Institute of Preventive Medicine, Denmark

## Abstract

**Background:**

In obesity, impaired adipose tissue function may promote secondary disease through ectopic lipid accumulation and excess release of adipokines, resulting in systemic low-grade inflammation, insulin resistance and organ dysfunction. However, several of the genes regulating adipose tissue function in obesity are yet to be identified.

**Methodology/Principal Findings:**

In order to identify novel candidate genes that may regulate adipose tissue function, we analyzed global gene expression in abdominal subcutaneous adipose tissue before and one year after bariatric surgery (biliopancreatic diversion with duodenal switch, BPD/DS) (n = 16). Adipose tissue from lean healthy individuals was also analyzed (n = 13). Two different microarray platforms (AB 1700 and Illumina) were used to measure the differential gene expression, and the results were further validated by qPCR. Surgery reduced BMI from 53.3 to 33.1 kg/m^2^. The majority of differentially expressed genes were down-regulated after profound fat loss, including transcription factors involved in stress response, inflammation, and immune cell function (e.g., FOS, JUN, ETS, C/EBPB, C/EBPD). Interestingly, a distinct set of genes was up-regulated after fat loss, including homeobox transcription factors (IRX3, IRX5, HOXA5, HOXA9, HOXB5, HOXC6, EMX2, PRRX1) and extracellular matrix structural proteins (COL1A1, COL1A2, COL3A1, COL5A1, COL6A3).

**Conclusions/Significance:**

The data demonstrate a marked switch of transcription factors in adipose tissue after profound fat loss, providing new molecular insight into a dichotomy between stress response and metabolically favorable tissue development. Our findings implicate homeobox transcription factors as important regulators of adipose tissue function.

## Introduction

Obesity has reached epidemic proportions and is associated with increased risk of type 2 diabetes, cardiovascular diseases, several forms of cancer, and other diseases [1]. As a potent endocrine organ as well as the body's primary energy storage reserve, adipose tissue plays key roles in systemic metabolism [2]. The pathogenic potential of adipose tissue is conferred by changes in morphology and cellular functions that involve local inflammation, aberrant hormonal signaling and adipokine secretion, and altered lipid storage dynamics [3], [4]. While adipocyte turnover appears to be tightly regulated [5], obesity may result from a combination of adipocyte hypertrophy and hyperplasia with varying contributions by these processes in different individuals [6]. Recent studies have indicated that hypertrophy may promote inflammation and dysfunctional adipose tissue via hypoxia and aberrant extracellular matrix remodeling [7], [8], [9]. Moreover, hypertrophy was found to be associated with a reduced adipocyte turnover [6]. It has been proposed that limited recruitment and development of adipocytes, and thereby reduced ability to expand the adipose tissue during energy surplus, may promote ectopic lipid accumulation and adverse effects on other organs [3], [10], [11].

Previous studies have demonstrated a marked increase in the expression of pro-inflammatory genes in adipose tissue in obesity [12], [13]. While isolated adipocytes show an increased inflammatory gene expression profile [14], increased infiltration and activation of non-fat cells, such as macrophages, may propagate local inflammation in adipose tissue [13], [15] and promote aberrant extracellular matrix remodeling [16]. Analyses of adipose tissue before and three months after gastric bypass surgery revealed a reduction in macrophages and altered expression of genes regulating inflammation and extracellular matrix [8], [17]. Moreover, adipose tissue gene expression is responsive to dietary intervention. Energy-restricted diets have been shown to improve the inflammatory profile [13] and to down-regulate genes involved in extracellular matrix [18] and production of polyunsaturated fatty acids [19]. It has also been demonstrated that dietary modification alters gene expression in adipose tissue irrespective of weight loss [20]. Interestingly, differences in adipose tissue gene regulation were observed depending on individual responses to caloric restriction [21].

Due to their profound fat loss, morbidly obese patients undergoing bariatric surgery represent a powerful model for studying changes in adipose tissue biology. Among the different forms of bariatric surgery, the biliopancreatic diversion with duodenal switch (BPD/DS) induces the most rapid and pronounced fat loss [22], and loss of excess fat is especially rapid within the first year of surgery [23]. Hence, we have analyzed global gene expression in subcutaneous abdominal adipose tissue (SAT) from 16 morbidly obese subjects before and one year after BPD/DS. To obtain a reference of normal expression values, we also included adipose tissue from 13 lean healthy controls undergoing inguinal hernia repair. The aim of the study was to identify novel candidate genes that may regulate adipose tissue function.

## Materials and Methods

### Ethics statement

Each enrolled subject gave their written consent after being informed about the study and legal rights. The study was approved by the Western Norway Regional Committee for Medical Research Ethics (REK).

### Subjects and adipose tissue biopsy

Anthropometric and biochemical data were recorded for patients before and one year after bariatric surgery (biliopancreatic diversion with duodenal switch) between 2003 and 2007. Biopsies of subcutaneous adipose tissue were collected from 16 patients (12 women) per-operatively and one year after surgery, and from 13 lean healthy control subjects who underwent inguinal hernia repair (6 women). Each biopsy was gently pressed flat in a sealable polyethylene bag, immediately transferred into liquid nitrogen, and stored at −80°C. Bariatric patients with Norwegian Caucasian origin and morbid obesity (BMI>45) were included. Average BMI was 53.3 kg/m^2^ (range 47 to 61) and average age was 39.3±10.9 (range 23 to 62 years). Fasting glucose, triglycerides, total-, HDL- and LDL cholesterol, insulin, insulin C-peptide, and CRP were measured in serum, and HbA_1C_ was measured in EDTA blood (Table 1). Significance values of anthropometric and biochemical data were calculated using paired-samples T-test. Seven of the bariatric patients were diabetic before surgery, and one patient continued on a low dose of insulin one year after surgery (Table S1). Inclusion criteria for the healthy lean control subjects were BMI<27 and no history of disorders or drug abuse. The control subjects had an average BMI of 23.0±2.48 (range 18.5 to 26.8) and an average age of 47.6±17.1 (range 20 to 77 years). All the measured fasting serum values of the control subjects (glucose, triglycerides, total-, HDL- and LDL cholesterol, insulin, insulin C-peptide) were within the normal range, except three CRP values (6, 8 and 18 mg/L). Homeostasis model assessment (HOMA) index, a measure of insulin resistance where a value of 1 is optimal, was calculated as fasting serum insulin (mIU/L)×glucose (mmol/L)/22.5.

**Table 1:** Anthropometric and biochemical data of 16 subjects before and one year after bariatric surgery (BPD/DS).

n = 16 (12 women)	Mean pre	Mean post	Mean	p-value
	surgery ± SD	surgery ± SD	change ± SD	(change)
Body weight (kg)	155.4±24.4	96.3±19.5	59.1±19.6	<0.0001
BMI (kg/m^2^)	53.3±4.3	33.1±5.0	20.2±5.6	<0.0001
SBP (mmHg)	142.9±14.1	126.3±16.9	16.7±21.4	0.0071
DBP (mmHg)	82.3±12.4	76.6±7.2	5.7±14.5	0.1370
Glucose (mmol/L)	6.65±1.66	5.13±0.68	1.52±1.40	0.0009
HbA1C (percent)	6.81±1.68	5.01±0.61	1.80±1.42	0.0002
Cholesterol (mmol/L)	4.75±0.88	3.44±0.69	1.31±0.77	<0.0001
TG (mmol/L)	1.61±0.57	1.26±0.66	0.35±0.56	0.0292
HDL (mmol/L)	1.07±0.27	0.99±0.27	0.08±0.21	0.1710
LDL (mmol/L)	3.36±0.76	2.20±0.70	1.16±0.67	<0.0001
Insulin (mIU/L)	27.55±18.95	6.39±5.60	21.16±17.66	0.0004
C-peptide (nmol/L)	1.29±0.70	1.00±1.09	0.29±1.36	0.4180
CRP (mg/L)	18.27±11.98	3.47±2.61	14.80±11.20	<0.0001

BMI, body mass index; SBP, systolic blood pressure; DBP, diastolic blood pressure; TG, triglycerides; HDL, high-density lipoprotein; LDL, low-density lipoprotein; C-peptide, Insulin C-peptide; CRP, C-reactive protein.

### Randomization of samples

To avoid systematic bias due to batch/lot and processing variability, bariatric surgery samples and control samples were balanced and randomized in each of the following steps: adipose tissue homogenization, RNA extraction, labeling, and microarray hybridization. Pre- and post-surgery pairs of adipose tissue samples were handled simultaneously.

### Homogenization and RNA extraction

Approximately 200–300 mg of frozen adipose tissue was sliced on dry ice and transferred to 2 ml microcentrifuge tubes with round bottom (Eppendorf). One ml lysing buffer (Qiazol, Qiagen) and a 5 mm metal bead (Millipore) were added and immediately followed by homogenization in a TissueLyser (Qiagen), with shaking three times for 2 minutes at a frequency of 25 Hz. The lysates were stored at −80°C. RNA from the adipose tissue was extracted using the RNeasy Lipid Tissue Midi Kit (Qiagen) according to the manufacturer protocol, and samples were treated with the RNase-Free DNase Set (Qiagen). Amount and quality of the extracted RNA were measured by the NanoDrop® ND-1000 spectrophotometer (NanoDrop Technologies, USA) and the Agilent 2100 Bioanalyzer (Agilent Technologies, USA).

### Illumina iScan system

A pilot microarray study of adipose tissue from 9 of the patients before and after surgery was performed using the Applied Biosystems (AB) 1700 Expression Array system (Text S1). The main microarray experiment was performed using the Illumina iScan, which is based upon fluorescence detection of biotin-labeled cRNA. Using the Illumina TotalPrep RNA Amplification Kit (version 280508, Applied Biosystems/Ambion, USA), 300ng of total RNA from each sample was reversely transcribed, amplified and Biotin-16-UTP–labeled. The amount (15–52 µg) and quality of labeled cRNA were measured using both the NanoDrop spectrophotometer and Agilent 2100 Bioanalyzer. 750ng of biotin-labeled cRNA was hybridized to the HumanRef-8 v.3 Illumina Sentrix BeadChip according to manufacturer's instructions. The HumanRef-8 v.3 BeadChip targets approximately 24,500 annotated RefSeq transcripts and covers 18,631 unique curated genes.

### Microarray data extraction and analysis

#### Quality control and preprocessing

Bead summary data was imported into GenomeStudio to remove control probes and to produce a text file containing the signal and detection p-values per probe for all samples. The text file was imported into J-Express Pro v.2.7, and signal intensity values were quantile normalized [24] and log transformed (base 2). Correspondence Analysis (CA) [25] and hierarchical clustering with Pearson Correlation as a distance measure were performed to look for global trends in the data. In the CA plot, the microarray data for genes and samples are projected onto a two-dimensional plane defined by the first and second principal components. The first principal component (along the x-axis) explains most of the total chi square, the second principal component explains second most of the total chi square. Samples that are close together in the plot have more similarity than samples further apart. The plots revealed sample Pre_11 as an outlier. Technical measures showed that this sample had lower yield from the labeling step than the other samples, and most of the signals were higher, including the P95, P05 and negative control signals. Therefore, the samples of patient 11 were removed from the dataset before further analysis.

#### Analysis of differentially expressed genes

Significance Analysis of Microarrays (SAM) [26] was used to look for differentially expressed genes. To obtain manageable datasets, differentially expressed genes were defined by fold change ≥1.5 and q-value = 0. Protein ANalysis THrough Evolutionary Relationships (PANTHER) (dated July 15^th^ 2009, www.pantherdb.org) was then used to search for over-represented functional categories among the differentially expressed genes. PANTHER describes genes in terms of biological processes, molecular functions, and pathways in hierarchies up to three levels. Each entry has an equivalent in Gene Ontology (www.geneontology.org). The Bonferroni correction for multiple testing was used in the calculation of PANTHER p-values.

The microarray data are MIAME compliant and are available in ArrayExpress with accession numbers E-TABM-862 (Illumina data) and E-TABM-864 (AB 1700 data).

### Validation of microarray data by qPCR

Ten genes were selected for validation by quantitative real-time RT-PCR (qPCR). The analysis was performed on a subset of the samples corresponding to the AB 1700 microarray analysis (pre- versus post-surgery, n = 9). cDNA synthesis was performed using the Transcriptor First Strand cDNA Synthesis Kit (Roche) (1 µg RNA per sample), followed by quantitative real-time RT-PCR with the LightCycler480 Probes Master kit and the LightCycler480 rapid thermal cycler system (Roche Applied Science). Target gene expression was quantified relative to the constitutive control gene TATA-binding protein (TBP) using specific Universal ProbeLibrary (UPL) probes and target-specific primers (Table S5). Target genes were amplified in duplex with TBP using the UPL Human TBP Gene Assay (Roche) with the exception of HOXA5, HOXB5, and IRX5 as a duplex run could not be performed for these genes. Instead, calculations were made with mean TBP values from five of the duplex runs. Efficiency of the transcripts was measured using standard curves with 1∶5 dilutions of an adipose tissue sample with concentrated cDNA. Negative controls were prepared by replacing the mRNA template with PCR-grade H_2_O. RT-PCR was performed according to the optimized protocol (Roche Applied Science). Fold change in mRNA expression was calculated pair-wise for each patient using the crossing point (CP) for each sample (triplicate) and the amplification efficiency for the transcript. The mRNA expressions were found to be non-linear and are presented as geometric mean with 95% confidence intervals (CI).

### Genomatix Gene2Promoter analysis

Genes whose promoters contain potential binding sites for homeobox transcription factors were identified using the *Gene2Promoter* software provided by Genomatix (http://www.genomatix.de/). Genes with a differential expression after bariatric surgery (152 up-regulated and 469 down-regulated) were input for the analysis. The software retrieved human gene promoters for each gene, and the data were filtered in order to extract genes with one or more binding sites for selected homeobox transcription factors.

## Results

### Clinical and biochemical characteristics

The average age of the bariatric patients before surgery and of the lean controls was 39.3±10.9 and 44.0±17.5 years, respectively. Average BMI of the bariatric patients was reduced by 20.2 kg/m^2^ one year after surgery (Table 1). Average BMI of the lean control subjects was 23.0 kg/m^2^. As expected, there was an improvement in glucose homeostasis and cardiovascular risk profile (Table 1). Six of the 16 bariatric patients had clear indications of manifest diabetes before surgery, assessed by fasting glucose values and diabetes history. Four of the patients were taking exogenous insulin prior to the surgery, and only one continued on a low dose after surgery (Table S1). Comparison of the average homeostasis model assessment (HOMA) index of the patients before and after surgery (8.6±6.41 versus 1.5±1.33, p-value = 0.0003) indicated a marked reversal of insulin resistance. Average HOMA index of the control subjects was 1.3±0.65. Before surgery there was sign of a low-grade inflammation evaluated by C-reactive protein (CRP), and this was reversed after fat loss.

### Marked shift in global gene expression after fat loss

**Figure 1:**
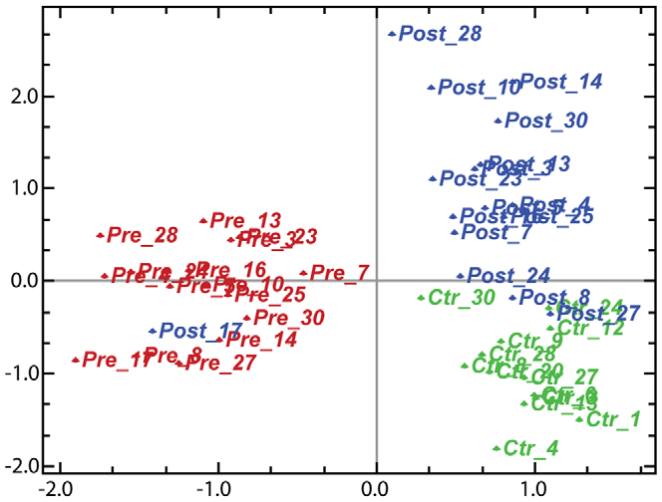
Correspondence Analysis showing projection of samples before and after bariatric surgery and lean healthy controls. The first principal component (10.69% component variance) separated the pre-operative samples from the post-operative and control samples, and the second principal component (6.36% component variance) separated the post-operative from control samples. The post-operative sample of patient 17 was similar to the pre-operative samples, suggesting an overall unaltered gene expression pattern after surgery for this patient. A technical error may have occurred, but we cannot rule out a unique gene expression pattern in the adipose tissue of this patient.

Microarray analysis revealed a substantial and highly consistent change in adipose tissue gene expression after surgery. Correspondence Analysis [25], which projects the differences in global gene expression in a two-dimensional plot, showed a clear-cut separation of the samples from the three groups (pre-surgery, post-surgery, and controls) (Figure 1). The first principal component, representing the largest component variance in the dataset (10.69%), showed that all the pre-operative samples grouped together, clearly separated from the post-operative and control samples. One exception was the post-operative sample of patient 17, which grouped together with the pre-operative samples. The second principal component, representing the second largest component variance in the dataset (6.36%), revealed that the control samples grouped together separately from the post-surgery samples.

Fat loss overall resulted in a down-regulation of genes in adipose tissue. Among the 621 differentially expressed genes using Illumina microarrays, 469 genes were down-regulated and 152 were up-regulated after surgery (Dataset S1). Strikingly, ninety-six of the 100 most differentially expressed genes showed a reduced expression after fat loss. Examples of strongly down-regulated genes included IL6 (Entrez Gene 3569), IL8 (Entrez Gene 3576), IL1B (Entrez Gene 3553), CCL2/MCP1 (Entrez Gene 6347), SOCS3 (Entrez Gene 9021), HIF1A (Entrez Gene 3091), PTGS2/COX-2 (Entrez Gene 5743) and NAMPT/VISFATIN (Entrez Gene 10135) (Table S2), which have previously been described in the context of obesity. Well-known genes involved in lipid metabolism were among the most differentially expressed single genes post-operatively, including LDLR (Entrez Gene 3949) and CH25H (Entrez Gene 9023) (down-regulated) and APOE (Entrez Gene 348), APOC1 (Entrez Gene 341) and SREBF1/SREBP-1c (Entrez Gene 6720) (up-regulated) (Table S2).

To test whether the use of medication or the presence of diabetes might affect the results, we analyzed the data excluding subjects on medication or with diabetes (n = 7). We found similar fold change values as when including all subjects, showing no clear effects of diabetes or medication on the global gene expression (data not shown).

### Switch from immunity and defense to developmental processes

**Figure 2:**
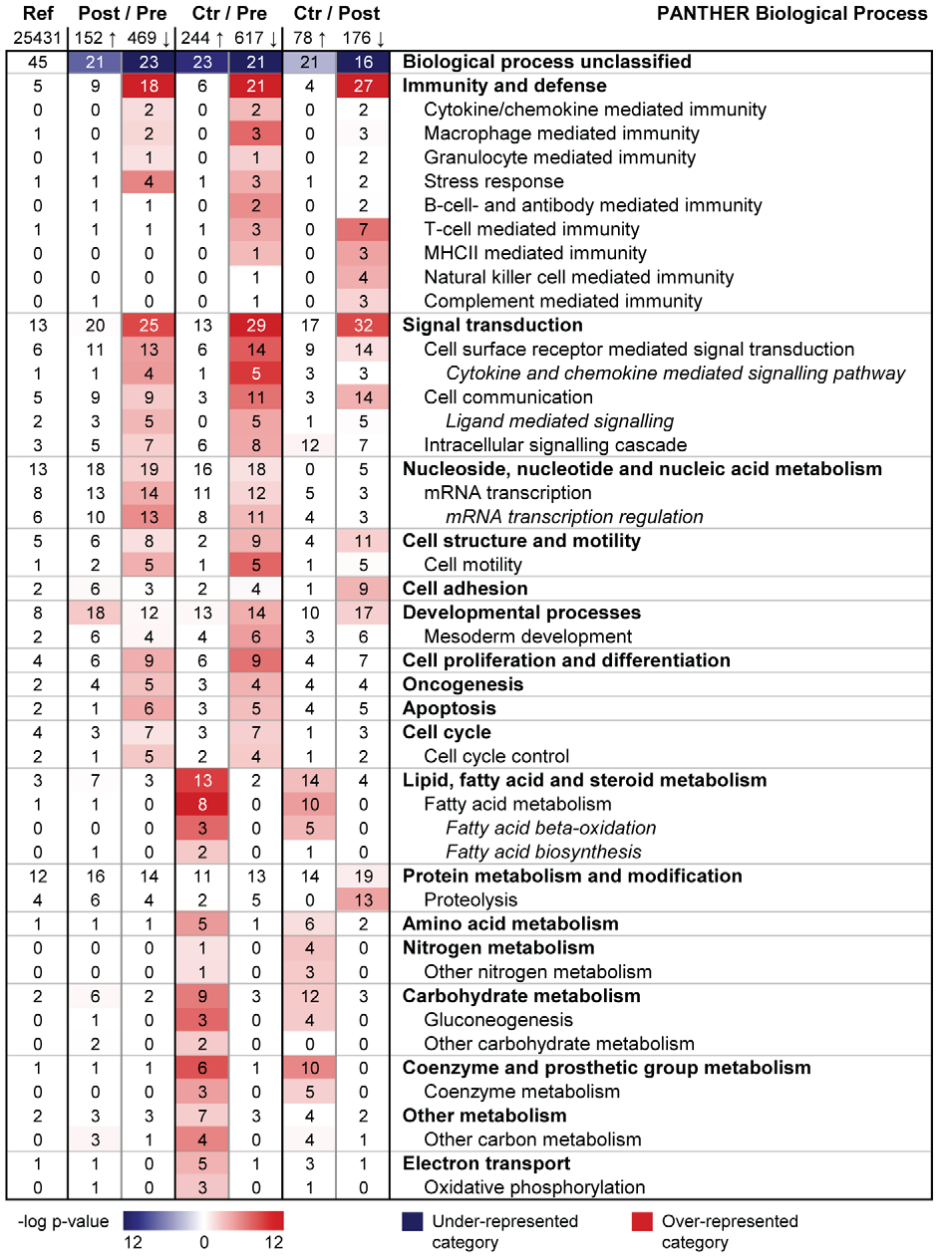
Functional categorization of differentially expressed genes in subcutaneous adipose tissue after fat loss (Biological Process). PANTHER was used to search for over-represented Biological Process categories among the most differentially expressed genes (q-value = 0, fold change ≥1.5), comparing adipose tissue before versus after bariatric surgery (n = 16) and versus controls (n = 13). The color intensity displays the statistical significance (−log p-value) of over- and under-represented PANTHER functional categories. A p-value<0.01 with Bonferroni correction for multiple testing was used as inclusion criterion for categories. Numbers presented in the table indicate the percentage of genes within a gene set that map to the given category, e.g. 18% of the 469 down-regulated genes map to the biological process ‘Immunity and defense’. The first column states the overall distribution of a term among all human NCBI genes (25,431), e.g. 5% of the genes are expected to map to ‘Immunity and defense’, hence this category is significantly over-represented among the down-regulated genes. Ref, reference (based on all human NCBI genes); Pre, pre-surgery biopsies; Post, post-surgery biopsies; Ctr, biopsies of lean controls; Arrow up, up-regulated/more expressed genes; Arrow down, down-regulated/less expressed genes.

Next, we searched for over-represented functional categories among the most differentially expressed genes using Protein ANalysis THrough Evolutionary Relationships (PANTHER) (dated July 15^th^ 2009, www.pantherdb.org). We found that the PANTHER Biological Process functional categories Immunity and defense and Signal transduction were strongly over-represented among down-regulated genes post-surgery (Figure 2). Over-represented sub-categories in the Immunity and defense category included Cytokine/chemokine mediated immunity, Macrophage mediated immunity, Granulocyte mediated immunity, and Stress response. Signal transduction sub-categories included Cell surface mediated signal transduction, Cytokine and chemokine mediated signaling pathway, and Cell communication. Interestingly, the analysis of up-regulated genes identified Developmental processes as the only PANTHER Biological Process category that was significantly over-represented post-operatively (Figure 2).

**Figure 3:**
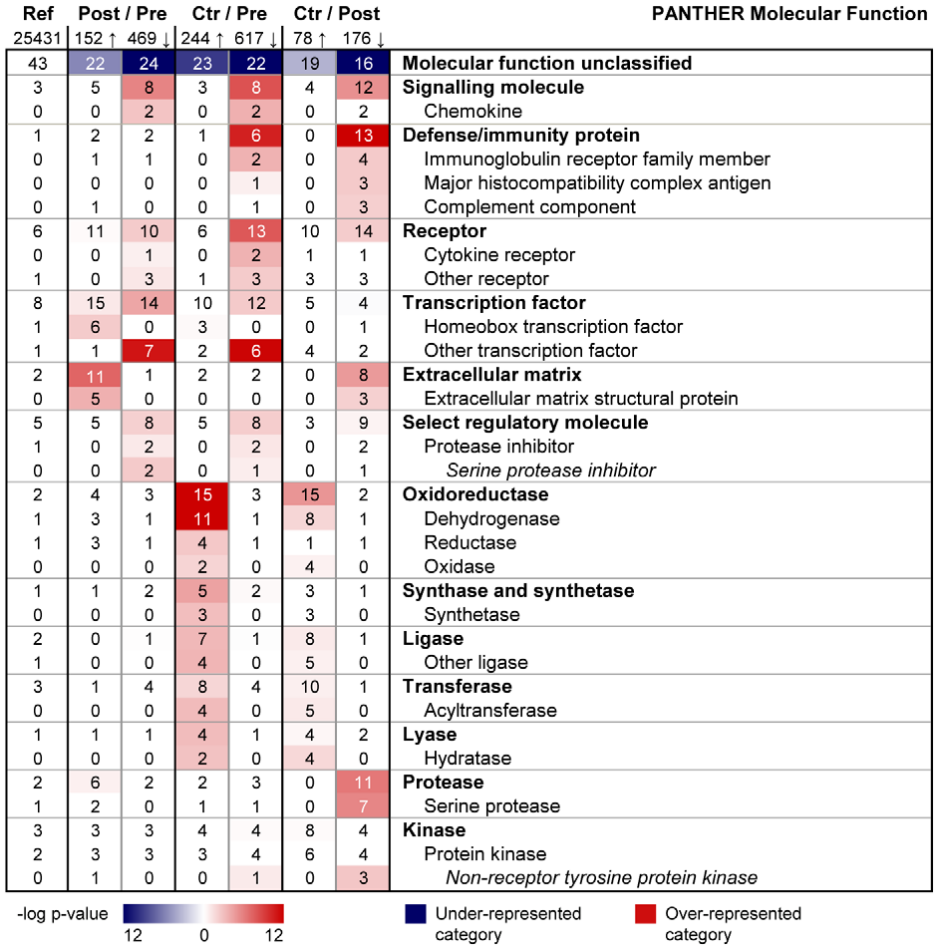
Functional categorization of differentially expressed genes in subcutaneous adipose tissue after fat loss (Molecular Function). PANTHER was used to search for over-represented Molecular Function categories among the most differentially expressed genes (q-value = 0, fold change ≥1.5), comparing adipose tissue before versus after bariatric surgery (n = 16) and versus controls (n = 13). The color intensity displays the statistical significance (−log p-value) of over- and under-represented PANTHER functional categories. A p-value<0.01 with Bonferroni correction for multiple testing was used as inclusion criterion for categories. Numbers presented in the table indicate the percentage of genes within a gene set that map to the given category, e.g. 8% of the 469 down-regulated genes map to the molecular function ‘Signalling molecule’. The first column states the overall distribution of a term among all human NCBI genes (25,431), e.g. 3% of the genes are expected to map to ‘Signalling molecule’, hence this category is significantly over-represented among the down-regulated genes. Ref, reference (based on all human NCBI genes); Pre, pre-surgery biopsies; Post, post-surgery biopsies; Ctr, biopsies of lean controls; Arrow up, up-regulated/more expressed genes; Arrow down, down-regulated/less expressed genes.

Over-represented PANTHER Molecular Function categories post-operatively included Signalling molecule, Receptor, Transcription factor, and Extracellular matrix (Figure 3). Genes belonging to Signalling molecule, Receptor, and Transcription factor were strongly over-expressed pre-surgery compared to post-operation and controls. Interestingly, our data revealed a switch in the expression of transcription factors, from a high expression of stress response related factors such as FOS (Entrez Gene 2353), JUN (Entrez Gene 3725), CEBPB (Entrez Gene 1051), CEBPD (Entrez Gene 1052), ETS1 (Entrez Gene 2113) and ETS2 (Entrez Gene 2114) in the morbidly obese state, to an increased expression of homeobox genes after surgery (Table 2 and 3, Table S3). The expression levels of homeobox genes (IRX3 (Entrez Gene 79191), IRX5 (Entrez Gene 10265), HOXA5 (Entrez Gene 3202), HOXA9 (Entrez Gene 3205), HOXB5 (Entrez Gene 3215), HOXC6 (Entrez Gene 3223), EMX2 (Entrez Gene 2018)) after surgery approximated the expression levels in healthy controls, except PRRX1 (Entrez Gene 5396) which was expressed at a higher level in post-surgery samples than in controls (Table 3). Finally, we found considerably higher expression levels of extracellular matrix genes (e.g. COL1A1 (Entrez Gene 1277), COL1A2 (Entrez Gene 1278), COL3A1 (Entrez Gene 1281), COL5A1 (Entrez Gene 1289), COL6A3 (Entrez Gene 1293)) post-surgery, whereas the expression levels were more similar in control and pre-surgery biopsies (Table 4, Table S3).

**Table 2:** Down-regulated genes in adipose tissue after profound fat loss (PANTHER category Other transcription factor, q-value = 0).

		Signal Intensity			FC
Gene	Definition	Pre	Post	Ctr	Post/Pre
BATF3	basic leucine zipper transcription factor, ATF-like 3	418	209	179	−1.98
CEBPB	CCAAT/enhancer binding protein (C/EBP), beta	12662	6983	6217	−1.83
CEBPD	CCAAT/enhancer binding protein (C/EBP), delta	5840	1917	2094	−3.23
ELF1	E74-like factor 1 (ets domain transcription factor)	2180	1425	1141	−1.52
ETS1	v-ets erythroblastosis virus E26 oncogene homolog 1	3414	2113	2059	−1.61
ETS2	v-ets erythroblastosis virus E26 oncogene homolog 2	1782	632	561	−3.06
FOS	v-fos	12716	1193	1196	−24.77
FOSB	FBJ murine osteosarcoma viral oncogene homolog B	25252	2392	970	−53.39
FOSL1	FOS-like antigen 1	509	186	162	−2.59
FOSL2	FOS-like antigen 2	308	192	187	−1.60
FOXC1	forkhead box C1	2787	1119	942	−2.63
IFI16	interferon, gamma-inducible protein 16	2630	1382	857	−1.84
IRF1	interferon regulatory factor 1	3073	864	636	−3.64
IRF7	interferon regulatory factor 7, transcript variant b	852	430	346	−2.03
JUN	jun oncogene	8097	1773	1496	−5.20
JUNB	jun B proto-oncogene	2005	319	236	−7.58
JUND	jun D proto-oncogene	20307	9053	9233	−2.33
LITAF	lipopolysaccharide-induced TNF factor	4585	2879	2871	−1.63
MAFF	v-maf (avian), transcript variant 1	367	189	178	−1.93
MNDA	myeloid cell nuclear differentiation antigen	572	277	185	−2.06
NFATC1	nuclear factor of activated T-cells, transcript var 1	548	237	228	−2.28
NFE2	nuclear factor (erythroid-derived 2), 45kDa	411	185	153	−2.27
NFE2L2	nuclear factor (erythroid-derived 2)-like 2	2001	1351	1180	−1.50
NFIL3	nuclear factor, interleukin 3 regulated	1601	447	349	−3.90
RFX2	regulatory factor X, 2, transcript variant 2	317	199	202	−1.55
SERTAD1	SERTA domain containing 1	2745	565	448	−5.65
SMAD7	SMAD family member 7	354	228	268	−1.53
SPRY1	sprouty homolog 1 (Drosophila), transcript variant 1	6884	3739	2578	−1.85
SPRY4	sprouty homolog 4 (Drosophila)	665	385	350	−1.78
TEAD4	TEA domain family member 4, transcript variant 3	465	227	232	−1.98
TSC22D1	TSC22 domain family, member 1, transcript variant 2	1511	835	1022	−1.80
TSC22D2	TSC22 domain family, member 2	2082	783	591	−2.79

Post, post-surgery; Pre, pre-surgery; Ctr, lean healthy controls; FC, fold change (based on log-transformed data).

**Table 3:** Up-regulated genes in adipose tissue after profound fat loss (PANTHER category Developmental processes, q-value = 0).

		Signal Intensity			FC
Gene	Definition	Pre	Post	Ctr	Post/Pre
ANG	angiogenin, transcript variant 2	1266	2083	2577	1.65
BHLHB5	basic helix-loop-helix domain containing, class B, 5	265	444	246	1.64
CD34	CD34 antigen, transcript variant 2	1711	2698	2348	1.59
CRABP2	cellular retinoic acid binding protein 2	460	823	504	1.72
GSDML	gasdermin-like (GSDML), transcript variant 1	283	531	711	1.76
IGLL1	immunoglobulin lambda-like polypeptide 1, trans var 1	1228	5219	317	3.61
IL11RA	interleukin 11 receptor, alpha, transcript variant 1	1350	2177	1528	1.62
KIT	v-kit	344	640	376	1.78
PCDH18	protocadherin 18	1125	2690	2430	2.33
PRICKLE1	prickle homolog 1 (Drosophila)	364	599	472	1.65
THRA	thyroid hormone receptor, alpha, transcript variant 2	316	636	396	1.82
*Homeobox transcription factor*					
EMX2	empty spiracles homeobox 2	244	404	312	1.63
MEOX2	mesenchyme homeobox 2	223	335	365	1.54
HOXA5	homeobox A5	801	1853	1608	2.38
HOXA9	homeobox A9	217	506	445	2.29
HOXB5	homeobox B5	308	466	465	1.52
HOXC6	homeobox C6, transcript variant 1	1243	2290	1988	1.86
IRX3	iroquois homeobox 3	532	1196	1007	2.10
IRX5	iroquois homeobox protein 5	166	254	228	1.50
PRRX1	paired related homeobox 1, transcript variant pmx-1a	2418	4251	2120	1.76

Post, post-surgery; Pre, pre-surgery; Ctr, lean healthy controls; FC, fold change (based on log-transformed data).

**Table 4:** Up-regulated genes in adipose tissue after profound fat loss (PANTHER category Extracellular matrix, q-value = 0).

		Signal Intensity			FC
Gene	Definition	Pre	Post	Ctr	Post/Pre
CLEC3B*	C-type lectin domain family 3, member B	3084	5104	4686	1.58
COL1A1	collagen, type I, alpha 1	1607	9727	2847	6.25
COL1A2	collagen, type I, alpha 2	2648	10595	3999	4.22
COL3A1	collagen, type III, alpha 1	7101	13956	7696	2.11
COL5A1*	collagen, type V, alpha 1	1408	2346	1595	1.62
COL6A3	collagen, type VI, alpha 3, transcript variant 1	3409	5433	3349	1.60
COL6A3	collagen, type VI, alpha 3, transcript variant 3	5416	9027	5594	1.67
FLRT2	fibronectin leucine rich transmembrane protein 2	312	516	381	1.63
ISLR	immunoglobulin superfamily L-rich repeat, trans var 1	493	869	630	1.73
KIAA0644	KIAA0644 gene product	241	471	403	1.91
LRRC17*	leucine rich repeat containing 17, transcript variant 2	259	751	526	2.78
LRRC17*	leucine rich repeat containing 17, transcript variant 1	175	316	253	1.76
MMP23B	matrix metallopeptidase 23B	286	606	377	2.03
PCOLCE	procollagen C-endopeptidase enhancer	878	1906	1052	2.10
PODN*	Podocan	1805	2904	2148	1.62

*Also represented in the category Developmental processes.

Post, post-surgery; Pre, pre-surgery; Ctr, lean healthy controls; FC, fold change (based on log-transformed data).

### Validation of results by AB 1700 microarrays and qPCR

To validate the results, we compared the Illumina microarray analysis to the AB 1700 microarray analysis (n = 9). The AB 1700 data confirmed the results (Figure S1, Dataset S2). Significance Analysis of Microarrays (SAM) identified 710 genes that were differentially expressed, of which 499 were down-regulated and 211 were up-regulated after surgery. As in the Illumina analysis, close to all of the top 100 differentially expressed genes were down-regulated after surgery (three up-regulated). Moreover, we also performed qPCR on the eight most significantly up-regulated homeobox transcription factors, in addition to COL1A1 (most up-regulated gene) and COL6A3 (recently connected with obesity). The qPCR analysis showed highly consistent results to the microarray analyses (Table 5).

**Table 5:** Validation of selected genes by qPCR.

	Fold change (Post/Pre)			
	Illumina	AB 1700	qPCR	qPCR
	(n = 16)	(n = 9)	(n = 9)	95% CI
COL1A1	6.25	8.20	2.35	1.32–4.17
COL6A3var1+3	1.63	1.32	1.20	0.70–2.04
EMX2	1.63	3.18	1.92	1.53–2.40
HOXA5	2.38	1.05	2.07	1.65–2.60
HOXA9	2.29	1.94	2.23	1.62–3.07
HOXB5	1.52	3.28	2.03	1.46–2.82
HOXC6	1.86	1.23	1.93	1.63–2.29
IRX3	2.10	3.22	3.59	2.23–5.76
IRX5	1.50	2.76	2.41	1.64–3.53
PRRX1	1.76	1.45	1.53	1.20–1.96

Post, post-surgery; Pre, pre-surgery; Illumina, Illumina microarrays; AB 1700, AB 1700 microarrays; qPCR, quantitative real-time RT-PCR; CI, confidence interval.

### Altered metabolic processes in bariatric patients

Based on the strong down-regulation, and since surgery normalized the biochemical parameters, we asked whether the gene expression levels after surgery might be similar to lean controls despite a higher BMI (33 versus 23). Comparing pre-surgery samples to controls we found 861 differentially expressed genes (244 more expressed and 617 less expressed in control samples). PANTHER functional analysis showed a high degree of similarity to the pre- versus post-surgery comparison (Figure 2 and 3). Moreover, the comparison of post-surgery samples to controls revealed 254 differentially expressed genes (78 more expressed and 176 less expressed in control patient samples) (Dataset S3). Functional analysis showed a distinct over-representation post-operatively of the Immunity and defense sub-categories B-cell, T-cell, and natural killer cell mediated immunity, and also the category Proteolysis (Figure 2), suggesting that these processes were not normalized after surgery. Moreover, the expression of several genes involved in metabolic processes was higher in the lean controls, including genes categorized to Fatty acid metabolism, Amino acid metabolism, Carbohydrate metabolism, and Coenzyme and prosthetic group metabolism (Figure 2). Of particular note, genes encoding oxidoreductases, including dehydrogenases, were expressed at higher levels in controls than in post-surgery subjects (Figure 3, Table 6).

**Table 6:** Up-regulated genes in adipose tissue of lean healthy controls versus post-surgery subjects (Oxidoreductase, q-value = 0).

		Signal Intensity			FC
Gene	Definition	Pre	Post	Ctr	Ctr/Post
ALDH1L1	aldehyde dehydrogenase 1 family, member L1	666	845	1504	1.91
ALDH2	aldehyde dehydrogenase 2 family (mitochondrial)	5430	5705	9662	1.71
ASPH	aspartate beta-hydroxylase, transcript variant 1	824	558	877	1.62
CYB5A	cytochrome b5 type A (microsomal)	5401	7024	10780	1.55
ECHDC3	enoyl Coenzyme A hydratase domain containing 3	1638	1686	3745	2.31
ECHS1	enoyl Coenzyme A hydratase, short chain, 1 (mitoch)	3167	3433	5864	1.72
HADH	hydroxyacyl-Coenzyme A dehydrogenase/3-ketoacyl- coenzyme A thiolase/enoyl-Coenzyme A hydratase (trifunctional protein), alpha subunit	4908	6078	9840	1.63
MAOA	monoamine oxidase A	5771	6082	9997	1.69
PECR	peroxisomal trans-2-enoyl-CoA reductase	1063	1172	1911	1.66
PLOD2	procollagen-lysine, 2-oxoglutarate 5-dioxygenase 2, var1	604	378	676	1.75
PLOD2	procollagen-lysine, 2-oxoglutarate 5-dioxygenase 2, var2	4692	3101	4711	1.56
SC5DL	sterol-C5-desaturase-like	765	797	1527	1.89
SEPW1	selenoprotein W, 1	1451	1093	1675	1.56

Post, post-surgery; Ctr, lean healthy controls; FC, fold change (based on log-transformed data).

### Promoter analysis of potential homeobox target genes

To further analyze the potential functions of homeobox transcription factors in adipose tissue, we performed promoter analysis of the most differentially expressed genes pre- versus post-surgery. Between eight and 48% of the genes contained one or more homeobox binding sites, depending on the homeobox transcription factor (Table 7). Many individual genes contained binding sites for several of the homeobox transcription factors (Table S6). We also examined genes with homeobox binding sites that were enriched in specific functional categories. The analysis revealed a high number of genes with homeobox binding sites in the PANTHER Molecular Function categories Transcription factor, Extracellular matrix, Signaling molecule, Select regulatory molecule and Receptor (Table 8, Table S7 and S8).

**Table 7:** Number of differentially expressed genes with binding sites for selected homeobox transcription factors.

Homeobox	Up-Regulated	Down-Regulated
TF	Genes (152)	Genes (469)
EMX2	14 (9%)	40 (9%)
HOXA5	44 (29%)	116 (25%)
HOXA9	59 (39%)	224 (48%)
HOXB5	28 (18%)	75 (16%)
HOXC6	37 (24%)	102 (22%)
IRX3	34 (22%)	73 (16%)
IRX5	51 (34%)	145 (31%)
PRRX1	13 (9%)	36 (8%)
One or more	110 (72%)	344 (73%)

**Table 8:** Number of genes in over-represented PANTHER categories with binding sites for one or more of the homeobox transcription factors.

PANTHER Molecular Function	Up-regulated	Down-regulated
Molecular function unclassified	23 (68%)	108 (96%)
Transcription factor	17 (74%)	67 (99%)
Extracellular matrix	7 (44%)	6 (98%)
Signalling molecule	7 (100%)	37 (95%)
Select regulatory molecule	6 (74%)	35 (97%)
Receptor	5 (31%)	41 (84%)

* Percent of total number of genes in each category (Figure 3, Post/Pre).

## Discussion

In this study we have shown that profound fat loss substantially alters the global gene expression profile of adipose tissue. To our knowledge, this is the first microarray study on adipose tissue obtained from patients treated with BPD/DS, the most effective bariatric surgical procedure for fat loss [22]. The prospective design and paired analysis reduces potential effects of genetic individuality. Our analysis revealed a marked switch of transcription factor usage, from a high expression of factors such as FOS, JUN, CEBPB, CEBPD, ETS1 and ETS2 in the morbidly obese state, to an increased expression of homeobox genes after profound fat loss. This switch of transcription factor profiles was associated with a strong reduction in inflammation and an increase in collagen expression.

Improved treatment of obesity-related diseases requires a better characterization of molecular mechanisms that determine adipose tissue function. Transcription factors mediate cellular responses to environmental and physiologic stimuli and intracellular signaling cascades. The homeobox genes constitute a special group of highly conserved transcription factors, characterized by a common DNA binding motif, the homeodomain, usually comprising 60 amino acids. Transcription factors encoded by the homeobox genes are master regulators of developmental patterning and segment diversification along the antero-posterior axis in every animal [27]. As targets of endocrine stimuli, they also regulate a host of processes related to normal and abnormal tissue development and function in adults [28]. However, the downstream homeobox target genes are largely unknown [29]. Moreover, little is known about the functions of homeobox genes in relation to obesity, though it has been suggested that they affect processes that are integral to adipose tissue function, such as adipogenesis [30], [31]. A total of 235 homeobox genes were recently proposed to be functional, categorized into eleven classes subdivided in 102 families [32].

Global gene expression studies comparing subcutaneous and visceral adipose tissue have revealed depot-specific expression of homeobox genes [33], [34]. These observations have raised the possibility that differential homeobox gene expression contributes to an increased disease risk associated with visceral obesity. The present study shows that the expression of several homeobox genes is responsive to fat loss, shedding new light on a potentially important role of homeobox transcription in adipose tissue. However, it is unclear at this time whether and to what extent homeobox transcription factors may promote fat loss, improve adipose tissue function, and/or simply respond to changes in adipose tissue mass. In the latter scenario, homeobox transcription factors may mediate the induction of factors involved in adipose tissue remodeling. A role for homeobox genes in tissue regeneration and repair has been observed [35]. Conceivably, the suggested role for homeobox genes in fibroblast differentiation [36] and adipogenesis [30], [31] may also point towards an involvement in adipose tissue development and adipocyte turnover. Functional studies of homeobox genes in preadipocytes as well as in earlier adipocyte precursor cells may lead to important new knowledge about adipose tissue biology. Our promoter analysis points towards a role for homeobox transcription factors in regulating a host of other transcription factors and a variety of molecular functions. Moreover, our study highlights the possibility that homeobox transcription factors may mitigate inflammation, or that a low expression during obesity may predispose to inflammation because of a reduced capability of tissue repair. Such a protective role for homeobox genes was demonstrated in the lungs of Hoxa5 knock-out mice, which were characterized by a perpetuated infiltration of activated macrophages [37].

The extracellular matrix is integral to tissue biology, and alteration of its functions has been implicated in a host of chronic conditions including obesity [8], [18]. In the present study of adipose tissue we found a strong over-expression in morbid obesity of several fibrogenic factors, including macrophage-secreted factors [16], ETS transcription factors [38], hypoxia-inducible factor (HIF)-1A [9], plasminogen activator inhibitor (PAI)-1/SERPINE1 (Entrez Gene 5054) [39], tenascin C (TNC) (Entrez Gene 3371) [40], serum/glucocorticoid kinase (SGK) (Entrez Gene 6446) [41], and connective tissue growth factor (CTGF/CCN2) (Entrez Gene 1490) [38]. It has been proposed that the inflammation and dysfunction of adipose tissue in obesity results from hypoxia-mediated fibrosis, which may promote adipocyte necrosis during continued adipocyte expansion [9]. A possible link between adipose tissue collagen and metabolic health was demonstrated in collagen VI knock-out mice, which were protected from metabolic derangements despite a massive increase in fat mass [42]. In addition, a recent study found a positive correlation between adipose tissue COL6A3 mRNA and body-mass index in humans and suggested that increased collagen VI expression may promote adipose tissue inflammation [43]. Of note, although we found a significant mean increase in COL6A3 expression after fat loss, some patients showed a higher expression in obesity, suggesting some inter-individual variation. Nonetheless, our finding that collagen gene expression was reduced in obesity relative to normal controls and after profound fat loss appears to conflict with these studies, and with the proposed negative impact of adipose tissue collagen on metabolic health. It should be noted that a previous study found no reversal of adipose tissue fibrosis in morbidly obese patients three months after gastric bypass surgery [8], suggesting that metabolic health can improve despite adipose tissue fibrosis being irreversible. It is conceivable that adipose tissue fibrosis impairs tissue function only during energy surplus, which is unlikely in the post-operative and malabsorptive state. Moreover, excessive collagen deposition may mainly occur in the presence of chronic cellular stress such as hypoxia and inflammatory responses [44], [45]. Contrary to pro-inflammatory transcription factors, there is evidence from mice suggesting that homeobox transcription factors promote normal tissue repair, despite a substantial increase in collagen synthesis and deposition [46]. Taken together, it is possible that a switch from pro-inflammatory transcription factors to homeobox transcription factors enables a metabolically favorable remodeling of adipose tissue after profound fat loss.

The amount of down-regulated genes and the degree of down-regulation were not as high in a previous study of differential gene expression after Roux-en-Y gastric bypass [17], which may be explained by the longer duration of our study (one year versus three months). Importantly, we did not employ a very low calorie diet (VLCD) prior to surgery, and post-surgery biopsies were obtained when fat loss began to plateau. We believe that the profound fat loss of our subjects represents close to the largest change in adipose tissue biology that can be obtained with current obesity treatment. The marked down-regulation in the pilot study prompted us to determine whether gene expression may be reduced to a below-normal level after such profound fat loss. By also including lean controls in the main study, we found that most genes returned to a normal level post-surgery despite a higher BMI than in lean controls (33.1 versus 23.0 kg/m^2^). In this regard, it is interesting to note that insulin sensitivity measures in weight-stable bariatric patients one year post-surgery were closer to lean subjects than to weight-matched controls [47]. Also of note we found a normalization of local markers of insulin resistance in adipose tissue, such as SOCS3 and IRS1 [48], [49]. Furthermore, the inflammatory gene expression in adipose tissue of obese subjects after a 28-day VLCD was more similar to lean than obese subjects [13]. On the other hand, in the present study many genes involved in metabolic processes did not reach the same expression level post-surgery as in lean controls, suggesting an effect of the higher BMI or other factors in the post-surgery state. This observation encourages further study of the identified metabolic genes in the post-operative state, and weight-matched controls should be used.

We found some variation between the pilot and main study. The higher number of patients in the main study yielded a higher statistical significance, revealing over-representation of PANTHER categories such as Extracellular matrix structural protein, which in the pilot study did not meet the strict significance criterion (p-value<0.01 with Bonferroni correction). In the PANTHER analysis we used fold change ≥1.5 and q-value = 0 as inclusion criteria for differentially expressed genes. Several of the same homeobox genes were differentially expressed with fold change ≥1.5 in the pilot study as in the main study, though only IRX3, IRX5, HOXC9 (Entrez Gene 3225), HOXC10 (Entrez Gene 3226) and NANOG (Entrez Gene 79923) also met the q-value = 0 criterion (Table S4). Moreover, variation in fold change values in the two analyses may be ascribed to the different detection systems. The chemiluminescence based AB expression array system is more sensitive and covers a broader range of signal intensities than the fluorescence based Illumina system. The combination of two microarray platforms strengthens our database.

Alterations in adipose tissue gene expression after bariatric surgery may partly be due to changes in the cellular composition of the tissue. In this regard, it is noteworthy that we obtained biopsies by surgical excision. Surgical biopsy was demonstrated to be superior to the needle biopsy technique, as the latter incompletely extracts the stromal vascular fraction [50]. Stromal vascular cells include non-adipose cell types such as preadipocytes, macrophages and lymphocytes that are integral to adipose tissue biology and whose functions are altered in obesity [8], [15], [16], [17], [51], [52], [53]. The strong down-regulation of gene expression in our study may partly reflect a reduced number and/or activity of these cell types, in particular immune cells. On the other hand, the genes with an increased expression after surgery are more likely to be actively up-regulated in cells whose phenotypes are altered by profound fat loss, including adipocytes, preadipocytes, and fibroblasts.

In summary, a switch from stress response to increased expression of homeobox transcription factors may represent a mechanism whereby adipose tissue function improves after fat loss and is maintained in normal tissue. Our results encourage further study of the potential roles for homeobox genes in regulating adipose tissue function.

## Supporting Information

Table S1 Medication in 16 patients before and one year after bariatric surgery.
(0.01 MB PDF)

Table S2 Expression values of selected genes previously implicated in obesity, and that were differentially expressed in adipose tissue one year after bariatric surgery.
(0.01 MB PDF)

Table S3 Description of differentially expressed genes in adipose tissue before versus after bariatric surgery, belonging to the PANTHER functional categories Other transcription factor, Developmental Processes, Homeobox transcription factor and Extracellular Matrix.
(0.02 MB PDF)

Table S4 Differentially expressed homeobox transcription factors in adipose tissue.
(0.02 MB PDF)

Table S5 Forward and reverse primers and UPL probes used for qPCR.
(0.01 MB PDF)

Table S6 Down-regulated genes in adipose tissue after bariatric surgery that contain one or more binding sites for one or more homeobox transcription factors.
(0.03 MB PDF)

Table S7 Up-regulated genes in adipose tissue after bariatric surgery that contain one or more binding sites for one or more homeobox transcription factors.
(0.01 MB PDF)

Table S8 PANTHER categories enriched with differentially expressed genes that contain one or more binding sites for one or more homeobox transcription factors.
(0.02 MB PDF)

Text S1 Materials and methods (AB 1700 Expression Array system)
(0.03 MB DOC)

Figure S1 Functional categorization of differentially expressed genes in adipose tissue after fat loss (AB 1700, n = 9). PANTHER was used to search for over-represented functional categories among the most differentially expressed genes (q-value = 0, fold change at least 1.5). The color intensity displays the statistical significance (−log p-value) of over- and under-represented PANTHER functional categories. Red color signifies an over-representation of genes mapping to a certain term, blue color an under-representation and white a representation as expected based on the overall distribution on the array. A p-value<0.01 was used as inclusion criterion for categories, with Bonferroni correction for multiple testing. Numbers presented in the table indicate the percentage of genes within a gene set that map to the given category, e.g. 18% of the 499 down-regulated genes map to the biological process ‘Immunity and defense’. The first column states the overall distribution of a term among all human NCBI genes (25,431), e.g. 5% of the genes are expected to map to ‘Immunity and defense’, hence this category is significantly over-represented among the down-regulated genes. Of note, unlike the Illumina data, the data showed an up-regulation of genes involved in B-cell and antibody-mediated immunity (e.g. immunoglobulins). However, the majority of these genes had records that were discontinued in the Entrez Gene database or were listed as hypothetical proteins. Ref, reference (based on all human NCBI genes); Pre, pre-surgery biopsies; Post, post-surgery biopsies; Ctr, lean controls; Arrow up, up-regulated/more expressed genes; Arrow down, down-regulated/less expressed genes (e.g. arrow up in Ctr/Post signifies higher expression in Ctr).
(0.48 MB TIF)

Dataset S1 Illumina microarray analysis of adipose tissue before versus one year after bariatric surgery.
(7.15 MB XLS)

Dataset S2 AB 1700 microarray analysis of adipose tissue before versus one year after bariatric surgery.
(8.84 MB XLS)

Dataset S3 Illumina microarray analysis of adipose tissue comparing morbidly obese and lean subjects.
(7.22 MB XLS)
