# Supplementary material for: Switch from Stress Response to Homeobox Transcription Factors in Adipose Tissue After Profound Fat Loss
Source: PLoS One. 2010 Jun 9;5(6):e11033. doi: 10.1371/journal.pone.0011033 (PMC2882947; doi:10.1371/journal.pone.0011033)
Supplement: Table S1 — Medication in 16 patients before and one year after bariatric surgery. (0.01 MB PDF) [file pone.0011033.s001.pdf]

TABLE S1 Medication in 16 patients before and one year after bariatric surgery (BPD/DS).

Patient	Pre/post surgery	Simvastatin (mg/day)	Antihypertensiva (number per day)	Metformin (mg/day)	Insulin (IU/day)	Diabetes mellitus
3	Pre	0	0	1000	0	DM
	Post	0	0	0	0	
4	Pre	0	0	0	0	
	Post	0	0	0	0	
5	Pre	0	0	0	0	
	Post	0	0	0	0	
7	Pre	40	3	1500	140	DM
	Post	0	2	0	28	
8	Pre	0	0	0	0	
	Post	0	0	0	0	
10	Pre	0	0	0	150	DM
	Post	0	0	0	0	
13 (male)	Pre	40	3	2000	0	DM
	Post	0	0	0	0	
14 (male)	Pre	40	2	0	0	
	Post	0	0	0	0	
16 (male)	Pre	40	2	1500	96	DM
	Post	0	1	0	0	
17	Pre	0	0	0	0	
	Post	0	0	0	0	
23	Pre	0	0	0	0	DM
	Post	0	0	0	0	
24	Pre	0	4	2000	140	DM
	Post	0	1	0	0	
25	Pre	0	0	0	0	
	Post	0	0	0	0	
27 (male)	Pre	0	0	0	0	
	Post	0	0	0	0	
28	Pre	0	0	0	0	
	Post	0	0	0	0	
30	Pre	0	0	0	0	
	Post	0	0	0	0	
