# Supplementary material for: Switch from Stress Response to Homeobox Transcription Factors in Adipose Tissue After Profound Fat Loss
Source: PLoS One. 2010 Jun 9;5(6):e11033. doi: 10.1371/journal.pone.0011033 (PMC2882947; doi:10.1371/journal.pone.0011033)
Supplement: Table S2 — Expression values of selected genes previously implicated in obesity, and that were differentially expressed in adipose tissue one year after bariatric surgery. (0.01 MB PDF) [file pone.0011033.s002.pdf]

TABLE S2 Expression values of selected genes previously implicated in obesity, that were differentially expressed in adipose tissue one year after bariatric surgery. Positive fold change (FC) values (Post/Pre) refer to up-regulation and negative values (Pre/Post) to down-regulation after bariatric surgery (q-value = 0).

Paired analysis, Illumina n=16, AB 1700 n=9 (entries in *italics*)

			**Signal intensity**			**FC**
**Symbol**	**Name**	**PANTHER Biological Processes**	**Pre**	**Post**	**Ctr**	**Post/Pre**
APOC1	Apolipoprotein C 1	Lipid and fatty acid transport; Transport	539<br>*12905*	2191<br>*70729*	640	3.21<br>*5.02*
APOE	Apolipoprotein E	Lipid and fatty acid transport; Transport	2320<br>*16002*	8716<br>*82398*	5719	3.53<br>*5.01*
CCL2	Chemokine (C-C motif) ligand 2 (MCP-1)	Cytokine and chemokine mediated signaling pathway; Ligand-mediated signaling; Cytokine/chemokine mediated immunity	13655<br>*460142*	2410<br>*41002*	926	-8.07<br>*-11.41*
CDKN1A	Cyclin-dependent kinase inhibitor 1A (p21)	Cell cycle control; Cell proliferation and differentiation; Tumor suppressor	15951<br>*164521*	2423<br>*9087*	839	-10.07<br>*-17.47*
CH25H	Cholesterol 25-hydroxylase	Cholesterol metabolism	1020<br>*26156*	196<br>*1308*	155	-5.57<br>*-20.36*
HIF1A	Hypoxia-inducible factor 1 alpha	mRNA transcription regulation; Neurogenesis	869<br>12276	399<br>3704	323	-2.10<br>*-3.09*
IL1B	Interleukin 1 beta	Cytokine and chemokine mediated signaling pathway; Ligand-mediated signaling; Macrophage-mediated immunity; Cytokine/chemokine mediated immunity; Apoptosis; Cell cycle control; Cell proliferation and differentiation	935<br>*15866*	209<br>*767*	157	-4.76<br>*-21.27*
IL6	Interleukin 6	Cytokine and chemokine mediated signaling pathway; MAPKKK cascade; JNK cascade; JAK-STAT cascade; Ligand-mediated signaling; Immunity and defense; Inhibition of apoptosis	5973<br>*139367*	638<br>*723*	211	-25.68<br>*-220.70*
IL8	Interleukin 8 (CXCL4)	Cytokine and chemokine mediated signaling pathway; Calcium mediated signaling; NF-kappaB cascade; Ligand-mediated signaling; T-cell mediated immunity; Macrophage-mediated immunity; Granulocyte-mediated immunity; Angiogenesis; Cell prolifer-ation and differentiation; Cell motility	7304<br>*44414*	789<br>*583*	211	-19.53<br>*-76.35*
IRS1	Insulin receptor substrate 1	MAPKKK cascade; Other intracellular signaling cascade; Glucose homeostasis	327<br>*5258*	524<br>*12356*	528	1.61<br>*2.35*
LDLR	Low density lipoprotein receptor	Oogenesis	8032<br>*57085*	1402<br>*2846*	773	-10.41<br>*-26.60*
NAMPT<br>*PBEF1*	Nicotinamide phosphoribosyl-transferase (Visfatin)	Ligand-mediated signaling; Immunity and defense	1825<br>*125739*	389<br>*11321*	348	-5.02<br>*-11.01*
OSM	Oncostatin M	Cytokine and chemokine mediated signaling pathway; JAK-STAT cascade; Ligand-mediated signaling; Immunity and defense; Developmental processes; Cell proliferation and differentiation; Other oncogenesis	521<br>*1287*	176<br>*215*	140	-3.05<br>*-4.87*

PLAUR	Plasminogen activator urokinase receptor (r-uPA)	Blood clotting	631	227	168	-2.86
			1432	407	225	-3.86
			25855	*3044*		*-7.17*
PTGS2	Prostaglandin-endoperoxide synthase 2 (COX-2)	Immunity and defense; Cell proliferation and differentiation	2534	303	228	-9.44
			20720	*736*		*-27.97*
SERPINE	Serpin peptidase inhibitor, clade E (PAI-1)	Proteolysis	1634	395	172	-5.62
			59227	*1524*		-31.28
SOCS3	Suppressor of cytokine signalling 3	JAK-STAT cascade;Inhibition of apoptosis; Developmental processes	448	173	155	-2.68
			18307	*497*		*-39.561*
SREBF1	Sterol regulatory binding factor 1 (SREBP-1)	Regulation of lipid, fatty acid and steroid metabolism; mRNA transcription	453	825	712	1.77
			4837	*12762*		*2.68*
THBS1	Thrombospondin 1	Blood clotting; Other developmental process	5898	1819	1506	-3.84
			78160	*16410*		*-4.97*
