# Supplementary material for: Switch from Stress Response to Homeobox Transcription Factors in Adipose Tissue After Profound Fat Loss
Source: PLoS One. 2010 Jun 9;5(6):e11033. doi: 10.1371/journal.pone.0011033 (PMC2882947; doi:10.1371/journal.pone.0011033)
Supplement: Table S3 — Description of differentially expressed genes in adipose tissue before versus after bariatric surgery, belonging to the PANTHER functional categories Other transcription factor, Developmental Processes, Homeobox transcription factor and Extracellular Matrix. (0.02 MB PDF) [file pone.0011033.s003.pdf]

**TABLE S3 Description of differentially expressed genes in adipose tissue before versus after bariatric surgery, belonging to the PANTHER functional categories Other transcription factor, Developmental Processes, Homeobox transcription factor and Extracellular Matrix**

| Symbol | Name | PANTHER Molecular Function | PANTHER Biological Process | Pathway |
|---|---|---|---|---|
| ANG | angiogenin, ribonuclease, Rnase A family, 5 (ALS9, HEL168, MGC22466, MGC71966, RNASE4, RNASE5) | Endoribonuclease<br>Other enzyme inhibitor<br>Hydrolase | RNA catabolism<br>Angiogenesis | |
| BATF3 | basic leucine zipper transcription factor, ATF-like 3<br>Jun dimerization protein p21SNFT (FLJ36352, FLJ37535, JDP1, JUNDM1) | Other transcription factor<br>Nucleic acid binding | Cell surface receptor mediated signal transduction | |
| BHLHB5 | basic helix-loop-helix domain containing, class B, 5 (BHLHB5, Beta3, CAGL85, TNRC20) | Basic helix-loop-helix transcription factor<br>Nucleic acid binding | mRNA transcription regulation<br>Other neuronal activity<br>Neurogenesis | |
| CD34 | CD34 molecule (RP11-328D5.2) | Cell adhesion molecule<br>Miscellaneous function | Cell adhesion<br>Hematopoiesis | |
| CEBPB | CCAAT/enhancer binding protein (C/EBP), beta (CRP2, IL6DBP, LAP, MGC32080, NF-IL6, TCF5) | Other transcription factor<br>Nucleic acid binding | mRNA transcription regulation<br>Stress response<br>Induction of apoptosis<br>Inhibition of apoptosis<br>Developmental processes<br>Cell proliferation and differentiation | |
| CEBPD | CCAAT/enhancer binding protein (C/EBP), delta (CELF, CRP3, NF-IL6-beta) | Other transcription factor<br>Nucleic acid binding | mRNA transcription regulation<br>Cell proliferation and differentiation | |
| CLEC3B | C-type lectin domain family 3, member B (DKFZp686H17246, TN, TNA) | Extracellular matrix structural protein | Skeletal development | |
| COL1A1 | collagen, type I, alpha 1 (OI4) | Extracellular matrix structural protein | Cell adhesion<br>Cell structure | Integrin signalling pathway->Collagen |
| COL1A2 | collagen, type I, alpha 2 (OI4) | Extracellular matrix structural protein | Cell adhesion<br>Cell structure | Integrin signalling pathway->Collagen |
| COL3A1 | collagen, type III, alpha 1 (EDS4A, FLJ34534) | Extracellular matrix structural protein | Cell adhesion<br>Cell structure | Integrin signalling pathway->Collagen |
| COL5A1 | collagen, type V, alpha 1 (RP11-263F14.1) | Extracellular matrix structural protein | Mesoderm development | Integrin signalling pathway->Collagen |
| COL6A3 | collagen, type VI, alpha 3 (DKFZp686D23123, DKFZp686K04147, DKFZp686N0262, FLJ34702, FLJ98399) | Extracellular matrix structural protein | Cell communication<br>Cell adhesion<br>Cell structure | Integrin signalling pathway->Collagen<br>Inflammation mediated by chemokine and cytokine signaling pathway->ExtraCellular matrix protein |

| | | | | |
|---|---|---|---|---|
| CRABP2 | cellular retinoic acid binding protein 2<br>(CRABP-II, RBP6) | Other transfer/carrier protein | Lipid and fatty acid transport<br>Lipid and fatty acid binding<br>Vitamin/cofactor transport<br>Steroid hormone-mediated signaling<br>Transport<br>Ectoderm development | |
| ELF1 | E74-like factor 1 (ets domain trans.factor) | Other transcription factor | mRNA transcription | PDGF signaling pathway->Ets |
| EMX2 | empty spiracles homolog 2 (Drosophila) | Homeobox transcription factor<br>Other DNA-binding protein | mRNA transcription regulation<br>Neurogenesis | |
| ETS1 | v-ets erythroblastosis virus E26<br>oncogene homolog 1 (avian)<br>(ETS-1, EWSR2, FLJ10768) | Other signaling molecule<br>Other transcription factor | mRNA transcription regulation<br>Signal transduction<br>Developmental processes<br>Cell proliferation and differentiation<br>Oncogenesis | Angiogenesis->v-Ets Avian<br>Erythroblastosis Virus E26 Oncogene<br>Homolog 1<br>PDGF signaling pathway->Ets<br>VEGF signaling pathway->v-Ets Avian<br>Ras Pathway->Ets |
| ETS2 | v-ets erythroblastosis virus E26<br>oncogene homolog 2 (avian)<br>(ETS2IT1) | Other signaling molecule<br>Other transcription factor | mRNA transcription regulation<br>Signal transduction<br>Developmental processes<br>Cell proliferation and differentiation<br>Oncogenesis | |
| FLRT2 | fibronectin leucine rich transmembrane<br>protein 2 (KIAA0405) | Receptor<br>Extracellular matrix | Cell surface receptor mediated signal<br>transduction | |
| FOS | v-fos FBJ murine osteosarcoma viral<br>oncogene homolog<br>(AP-1, C-FOS) | Other transcription factor<br>Nucleic acid binding | Cell surface receptor mediated signal<br>transduction | Insulin/IGF pathway-mitogen activated<br>protein kinase kinase/MAP kinase cascade-<br>>c-fos<br>PDGF signaling pathway->c-fos<br>Interleukin signaling pathway->c-fos<br>B cell activation->fos<br>T cell activation->fos<br>Huntington disease->Fos protein<br>Angiogenesis->c-Fos Transcription Factor<br>TGF-beta signaling pathway->Co-<br>activators or corepressors<br>Apoptosis signaling pathway->Fos |

| | | | | |
|---|---|---|---|---|
| FOSB | FBJ murine osteosarcoma viral oncogene homolog B (AP-1, DKFZp686C0818, G0S3, GOS3, GOSB, MGC42291) | Other transcription factor<br>Nucleic acid binding | Cell surface receptor mediated signal transduction | |
| FOSL1 | FOS-like antigen 1<br>(FRA, FRA1, fra-1) | Other transcription factor<br>Nucleic acid binding | Cell surface receptor mediated signal transduction | TGF-beta signaling pathway->Co-activators or corepressors |
| FOSL2 | FOS-like antigen 2<br>(FLJ23306, FRA2) | Other transcription factor<br>Nucleic acid binding | Cell surface receptor mediated signal transduction | |
| FOXC1 | forkhead box C1<br>(ARA, FKHL7, FREAC-3, FREAC3, IGDA, IHG1, IRID1, RIEG3) | Other transcription factor<br>Nucleic acid binding | Cell surface receptor mediated signal transduction | Insulin/IGF pathway-protein kinase B signaling cascade->Forkhead transcription factor<br>PI3 kinase pathway->FOXO<br>Interleukin signaling pathway->Forkhead in Rhabdomyosarcoma-like 1<br>TGF-beta signaling pathway->Co-activators or corepressors |
| GSDML | gasdermin-like<br>(GSDMB, PP4052, PRO2521) | Molecular function unclassified | Cell surface receptor mediated signal transduction | |
| HOXA5 | homeobox A5<br>(HOX1, HOX1.3, HOX1C, MGC9376) | Homeobox transcription factor<br>Nucleic acid binding | Cell surface receptor mediated signal transduction | Wnt signaling pathway->Wnt Target Genes |
| HOXA9 | homeobox A9 (ABD-B, HOX1, HOX1.7, HOX1G, MGC1934) | Homeobox transcription factor<br>Other DNA-binding protein | Cell surface receptor mediated signal transduction | |
| HOXB5 | homeobox B5<br>(HHO.C10, HOX2, HOX2A, HU-1, Hox2.1) | Homeobox transcription factor<br>Nucleic acid binding | Cell surface receptor mediated signal transduction | Wnt signaling pathway->Wnt Target Genes |
| HOXC6 | homeobox C6<br>(CP25, HHO.C8, HOX3, HOX3C) | Homeobox transcription factor<br>Nucleic acid binding | Cell surface receptor mediated signal transduction | Wnt signaling pathway->Wnt Target Genes |
| IFI16 | interferon, gamma-inducible protein 16<br>(IFNGIP1, MGC9466, PYHIN2) | Other transcription factor | Cell surface receptor mediated signal transduction | |
| IGLL1 | immunoglobulin lambda-like polypeptide 1<br>(14.1, CD179b, IGL1, IGL5, IGLJ14.1, IGLL, IGO, IGVPB, VPREB2) | Immunoglobulin | Cell surface receptor mediated signal transduction | |
| IL11RA | interleukin 11 receptor, alpha<br>(hCG_2011440, MGC2146) | Interleukin receptor | Cell surface receptor mediated signal transduction | Interleukin signaling pathway->Receptor subunit alpha |
| IRF1 | interferon regulatory factor 1<br>(IRF-1, MAR) | Other transcription factor<br>Nucleic acid binding | Cell surface receptor mediated signal transduction | |

| | | | | |
|---|---|---|---|---|
| IRF7 | interferon regulatory factor 7<br>(IRF-7H, IRF7A) | Other transcription factor<br>Nucleic acid binding | Cell surface receptor mediated signal transduction | Toll receptor signaling pathway->Interferon (INF) regulatory factor 3 |
| IRX3 | iroquois homeobox protein 3<br>(FLJ99187, IRX-1) | Homeobox transcription factor<br>Nucleic acid binding | Cell surface receptor mediated signal transduction | |
| IRX5 | iroquois homeobox protein 5<br>(IRX-2a, IRXB2) | Homeobox transcription factor<br>Nucleic acid binding | Cell surface receptor mediated signal transduction | |
| ISLR | immunoglobulin superfamily containing leucine-rich repeat (HsT17563, MGC102816) | Receptor<br>Extracellular matrix | Cell surface receptor mediated signal transduction | |
| JUN | jun oncogene<br>(AP-1, AP1, c-Jun) | Other transcription factor<br>Nucleic acid binding | Cell surface receptor mediated signal transduction | Toll receptor signaling pathway->activator protein-1<br>Inflammation mediated by chemokine and cytokine signaling pathway->Activator protein-1<br>Huntington disease->c-Jun<br>Apoptosis signaling pathway->c-Jun<br>Oxidative stress response->jun sarcoma virus 17 oncogene homolog<br>TGF-beta signaling pathway->Co-activators or corepressors<br>Angiogenesis->c-Jun Trans. Factor<br>FAS signaling pathway->c-Jun<br>PDGF signaling pathway->c-Jun<br>T cell activation->jun<br>Ras Pathway->AP1<br>B cell activation->jun |
| JUNB | jun B proto-oncogene<br>(AP-1) | Other transcription factor<br>Nucleic acid binding | Cell surface receptor mediated signal transduction | Inflammation mediated by chemokine and cytokine signaling pathway->Activator protein-1 |
| JUND | jun D proto-oncogene<br>(AP-1) | | | TGF-beta signaling pathway->Co-activators or corepressors |
| KIAA0644 | KIAA0644 gene product (unassigned) | Receptor<br>Extracellular matrix | Cell surface receptor mediated signal transduction | |
| KIT | v-kit Hardy-Zuckerman 4 feline sarcoma viral oncogene homolog<br>(C-Kit, CD117, PBT, SCFR) | Tyrosine protein kinase receptor<br>Protein kinase | Cell surface receptor mediated signal transduction | |

| | | | | |
|---|---|---|---|---|
| LITAF | lipopolysaccharide-induced TNF factor (FLJ38636, MGC116698, MGC116700, MGC116701, MGC125274, MGC125275, MGC125276, PIG7, SIMPLE, TP53I7) | Other transcription factor | Cell surface receptor mediated signal transduction | |
| LRRC17 | leucine rich repeat containing 17 (P37NB) | Receptor<br>Extracellular matrix | Cell surface receptor mediated signal transduction | |
| MAFF | v-maf musculoaponeurotic fibrosarcoma oncogene homolog F (avian) (CTA-447C4.1, U-MAF, hMafF) | Other transcription factor<br>Nucleic acid binding | Cell surface receptor mediated signal transduction | |
| MEOX2 | mesenchyme homeobox 2 (GAX, MOX2) | Homeobox transcription factor<br>Other DNA-binding protein | Cell surface receptor mediated signal transduction | |
| MMP23B | matrix metallopeptidase 23B (RP11-345P4.2, MIFR, MIFR-1, MMP22) | Metalloprotease<br>Other extracellular matrix | Cell surface receptor mediated signal transduction | Alzheimer disease-presenilin pathway->Matrix metalloprotease |
| MNDA | myeloid cell nuclear differentiation antigen (PYHIN3) | Other transcription factor | Cell surface receptor mediated signal transduction | |
| NFATC1 | nuclear factor of activated T-cells, cytoplasmic, calcineurin-dependent 1 (MGC138448, NF-ATC, NFAT2, NFATc) | Other transcription factor | Cell surface receptor mediated signal transduction | Wnt signaling pathway->NFAT<br>Inflammation mediated by chemokine and cytokine signaling pathway->Nuclear factor of activated T cells<br>B cell activation->NF-AT<br>T cell activation->NF-AT |
| NFE2 | nuclear factor (erythroid-derived 2), 45kDa (NF-E2, p45) | Other transcription factor<br>Nucleic acid binding | Cell surface receptor mediated signal transduction | |
| NFE2L2 | nuclear factor (erythroid-derived 2)-like 2 (NRF2) | Other transcription factor<br>Nucleic acid binding | Cell surface receptor mediated signal transduction | |
| NFIL3 | nuclear factor, interleukin 3 regulated (E4BP4, IL3BP1, NF-IL3A, NFIL3A) | Other transcription factor | Cell surface receptor mediated signal transduction | |
| PCDH18 | protocadherin 18 (DKFZp434B0923, KIAA1562, PCDH68L) | Cadherin | Cell surface receptor mediated signal transduction | Wnt signaling pathway->Cadherin<br>Cadherin signaling pathway->Cadherin |
| PCOLCE | procollagen C-endopeptidase enhancer (PCPE, PCPE1) | Other enzyme regulator<br>Other extracellular matrix | Cell surface receptor mediated signal transduction | |
| PODN | podocan (MGC24995, PCAN, SLRR5A) | Receptor<br>Extracellular matrix | Cell surface receptor mediated signal transduction | |

| | | | | |
|---|---|---|---|---|
| PRICKLE1 | prickle homolog 1 (Drosophila)<br>(EPM1B, FLJ31627, FLJ31937, MGC138902, MGC138903, RILP) | Actin binding cytoskeletal protein<br>Structural protein | Cell surface receptor mediated signal transduction | |
| PRRX1 | paired related homeobox 1<br>(PHOX1, PMX1, PRX1, rHOX, mHOX, k2) | Homeobox transcription factor<br>Other DNA-binding protein | Cell surface receptor mediated signal transduction | |
| RFX2 | regulatory factor X, 2 (influences HLA class II expression) (FLJ14226) | Other transcription factor | Cell surface receptor mediated signal transduction | |
| SERTAD1 | SERTA domain containing 1<br>(SEI1, TRIP-Br1) | Other transcription factor | Cell surface receptor mediated signal transduction | |
| SMAD7 | SMAD family member 7<br>(CRCS3, FLJ16482, MADH7, MADH8) | Other transcription factor | Cell surface receptor mediated signal transduction | TGF-beta signaling pathway->Inhibitory Smads<br>TGF-beta signaling pathway->Rsmads<br>TGF-beta signaling pathway->I-SMAD |
| SPRY1<br><br><br>SPRY4 | sprouty homolog 1, antagonist of FGF signaling (Drosophila)<br>(hSPRY1)<br>sprouty homolog 4 (Drosophila) | Other signaling molecule<br>Other transcription factor | Cell surface receptor mediated signal transduction | FGF signaling pathway->sprouty<br>EGF receptor signaling pathway->SPRY<br>EGF receptor signaling pathway->sprouty |
| TEAD4 | TEA domain family member 4<br>(EFTR-2, MGC9014, RTEF1, TCF13L1, TEF-3, TEF3, TEFR-1, hRTEF-1B) | Other transcription factor | Cell surface receptor mediated signal transduction | |
| THRA | thyroid hormone receptor, alpha (erythroblastic leukemia viral (v-erb-a) oncogene homolog, avian)<br>(AR7, EAR7, ERB-T-1, ERBA, ERBA1, MGC000261, MGC43240, NR1A1, THRA1, THRA2, c-ERBA-1) | Nuclear hormone receptor<br>Transcription factor | Cell surface receptor mediated signal transduction | |
| TSC22D1 | TSC22 domain family, member 1<br>(DKFZp686O19206, MGC17597, RP11-269C23.2, TGFB1I4, TSC22) | Other transcription factor | Cell surface receptor mediated signal transduction | |
| TSC22D2 | TSC22 domain family, member 2<br>(KIAA0669, TILZ4a, TILZ4b, TILZ4c) | Other transcription factor | Cell surface receptor mediated signal transduction | |
