# Supplementary material for: Switch from Stress Response to Homeobox Transcription Factors in Adipose Tissue After Profound Fat Loss
Source: PLoS One. 2010 Jun 9;5(6):e11033. doi: 10.1371/journal.pone.0011033 (PMC2882947; doi:10.1371/journal.pone.0011033)
Supplement: Table S4 — Differentially expressed homeobox transcription factors in adipose tissue. (0.02 MB PDF) [file pone.0011033.s004.pdf]

TABLE S4 Differentially expressed homeobox transcription factors in adipose tissue

A. Before versus one year after bariatric surgery (Illumina microarrays, paired SAM, n=16)

			Signal Intensity			FC	q-val
**Rank**	**Symbol**	**Name**	**Pre**	**Post**	**Ctr**	**Post/Pre**	
107	HOXC9	homeobox C9	185	251	258	1.36	0
116	HOXA9	homeobox A9	217	506	445	2.29	0
137	HOXA5	homeobox A5	801	1853	1608	2.38	0
268	HOXC6	homeobox C6, transcript variant 1	1243	2290	1988	1.86	0
309	EMX2	empty spiracles homeobox 2	244	404	312	1.63	0
386	IRX5	iroquois homeobox protein 5	166	254	228	1.50	0
421	MEIS2	Meis homeobox 2, transcript variant g	308	466	465	1.26	0
448	IRX3	iroquois homeobox 3	532	1196	1007	2.10	0
466	SIX5	SIX homeobox 5	513	742	742	1.45	0
515	HOXB5	homeobox B5	308	466	465	1.52	0
616	PRRX1	paired related homeobox 1, transcript variant pmx-1a	2418	4251	2120	1.76	0
952	HOXA3	homeobox A3, transcript variant 2	158	214	237	1.33	0
960	HLX	H2.0-like homeobox	456	286	266	-1.61	0
968	ZFHX4	zinc finger homeobox 4	238	327	263	1.38	0
981	HOXD12	homeobox D12	427	587	431	1.36	0
1226	NKX3-1	NK3 homeobox 1	252	167	169	-1.48	0
1518	MKX	mohawk homeobox	154	196	301	1.26	0
1673	HOXA6	homeobox A6	213	302	293	1.39	0
1787	GSC	goosecoid homeobox	160	187	200	1.17	0
1906	RHOXF1	Rhox homeobox family, member 1	235	188	218	-1.25	0
2047	HOXB2	homeobox B2	367	466	503	1.27	0
2084	HHEX	hematopoietically expressed homeobox	291	428	346	1.48	0
2229	HOXA2	homeobox A2	158	182	179	1.16	0
2385	HOXA4	homeobox A4	160	182	196	1.14	0
2534	MEOX2	mesenchyme homeobox 2	223	335	365	1.54	0
2650	SIX4	SIX homeobox 4	186	224	259	1.21	0
2826	ADNP	activity-dependent neuroprotector homeobox, trans var 2	410	496	515	1.20	0

SAM, Significance Analysis of Microarrays; Pre, before surgery; Post, after surgery; Ctr, lean healthy controls; FC, fold change; q-val, q-value.

B. Post-surgery (n=16) versus healthy controls (n=13) (Illumina microarrays, unpaired SAM, q-val < 1)

			Signal Intensity			FC	q-val
**Rank**	**Symbol**	**Name**	**Pre**	**Post**	**Ctr**	**Post/Ctr**	
25	HOXA10	homeobox A10, transcript variant 2	141	141	191	-1.35	0
26	PRRX1	paired related homeobox 1, transcript variant pmx-1a	2418	4251	2120	2.03	0
284	HOXA10	homeobox A10, transcript variant 1	455	537	754	-1.42	0
321	PRRX2	paired related homeobox 2	600	744	457	1.61	0
330	SATB2	SATB homeobox 2	272	246	308	-1.25	0
475	MKX	mohawk homeobox	154	196	301	-1.48	0
615	HOXD12	homeobox D12	427	587	431	1.36	0
1257	ZFHX4	zinc finger homeobox 4	238	327	263	1.25	0.166
1482	HOXA11	homeobox A11	141	139	154	-1.10	0.287
1592	TSHZ3	teashirt zinc finger homeobox 3	514	445	370	1.20	0.361
1872	EMX2	empty spiracles homeobox 2	244	404	312	1.29	0.613

SAM, Significance Analysis of Microarrays; Pre, before surgery; Post, after surgery; Ctr, lean healthy controls; FC, fold change; q-val, q-value.

C. Before versus one year after bariatric surgery (AB 1700 microarrays, paired SAM, n=9, q-val < 5)

			Signal Intensity		FC	q-val
**Rank**	**Symbol**	**Name**	**Pre**	**Post**	**Post/Pre**	
301	HOXC9	homeobox C9	4946	12923	2.61	0
462	IRX3	iroquois homeobox protein 3	4082	14092	3.22	0
505	IRX5	iroquois homeobox protein 5	926	2553	2.76	0
621	HOXC10	homeobox C10	4505	9448	2.07	0
685	NANOG	Nanog homeobox	457	1359	3.43	0
743	HOXB5	homeobox B5	1609	4376	3.28	0.076
758	HOXA6	homeobox A6	564	1189	2.08	0.076
848	HOXA7	homeobox A7	8230	16561	2.00	0.137
920	DUX2	double homeobox, 2	768	1694	2.15	0.137
1389	SIX5	sine oculis homeobox homolog 5 (Drosophila)	12258	22194	1.83	0.547
1761	HOXB6	homeobox B6	1536	2629	1.78	0.980
2088	HOXD4	homeobox D4	3032	4995	1.74	1.497
2482	HOXA9	homeobox A9	417	699	1.94	2.216
2846	MEOX2	mesenchyme homeobox 2	7590	10798	2.21	3.059
2871	DDB2|LHX3	damage-specific DNA binding protein 2|LIM homeobox 3	3377	5457	1.63	3.080
2874	HOXB2	homeobox B2	5459	8474	1.57	3.080
3302	HOXC8	homeobox C8	1768	2888	1.59	4.015
3707	HESX1	homeobox, ES cell expressed 1	446	663	1.81	4.950

SAM, Significance Analysis of Microarrays; Pre, before surgery; Post, after surgery; Ctr, lean healthy controls; FC, fold change; q-val, q-value.
