# Supplementary material for: Switch from Stress Response to Homeobox Transcription Factors in Adipose Tissue After Profound Fat Loss
Source: PLoS One. 2010 Jun 9;5(6):e11033. doi: 10.1371/journal.pone.0011033 (PMC2882947; doi:10.1371/journal.pone.0011033)
Supplement: Table S5 — Forward and reverse primers and UPL probes used for qPCR. (0.01 MB PDF) [file pone.0011033.s005.pdf]

TABLE S5 Forward and reverse primers and UPL probes used for qPCR.

Target gene	Forward (left) primer	Reverse (right) primer	UPL Probe	Duplex
COL1A1	5`-aggtccccctggaaagaa-3`	5`-aatcctcgagcaccctga-3`	#60	TBP
COL6A3var1+3	5`-cctaaccacatatgttagtggaggt-3`	5`-gaatgtctcgcttgctctctg-3`	#69	TBP
EMX2	5`-aggaagcagctggcacac-3`	5`-tcttcggttctgaaaccatactt-3`	#30	TBP
HOXA5	5`-gcgcaagctgcacataag-3`	5`-cggttgaagtggaactcctt-3`	#1	
HOXA9	5`-aaaacaatgctgagaatgagagc-3`	5`-tataggggcaccgcttttt-3`	#66	TBP
HOXB5	5`-aagcttcacatcagccatga-3`	5`-cggttgaagtggaactccttt-3`	#1	
HOXC6var1	5`-cagaaccggatctactcgactc-3`	5`-ccggctggaactgaacac-3`	#2	TBP
IRX3	5`-aaaagttactcaagacagctttcca-3`	5`-ggatgaggagagagccgata-3`	#57	TBP
IRX5	5`-gacctggagaagaacgacga-3`	5`-gccttctgctcagctcctc-3`	#45	
PRRX1	5`-gtggagcagcccatcgta-3`	5`-tgggagggacgaggatct-3`	#15	TBP

UPL, Universal ProbeLibrary (Roche Applied Science)
