# Supplementary material for: Switch from Stress Response to Homeobox Transcription Factors in Adipose Tissue After Profound Fat Loss
Source: PLoS One. 2010 Jun 9;5(6):e11033. doi: 10.1371/journal.pone.0011033 (PMC2882947; doi:10.1371/journal.pone.0011033)
Supplement: Table S6 — Down-regulated genes in adipose tissue after bariatric surgery that contain one or more binding sites for one or more homeobox transcription factors. (0.03 MB PDF) [file pone.0011033.s006.pdf]

TABLE S6 Down-regulated genes in adipose tissue after bariatric surgery that contain one or more binding sites for one or more homeobox transcription factors

Target gene	Post/Pre	Potential regulators
ABL2	-1.94	HOXA5, HOXA9, IRX3, IRX5
ACSL4	-1.5	HOXA9, IRX5
ADAM19	-1.55	HOXA5, HOXA9, IRX3
ADAMTS1	-8.35	IRX5
ADAMTS9	-3.31	HOXA5, HOXA9, HOXB5, HOXC6, IRX3, IRX5
ADFP	-1.86	HOXA9, HOXB5
AHR	-1.6	HOXA5, HOXA9, HOXC6, IRX3, PRRX1
AKAP12	-1.91	HOXB5
AKR1C2	-1.55	HOXA9
ALAS2	-2.91	HOXA5, HOXA9, IRX3, IRX5
ALDH1A2	-1.54	HOXA9, IRX5, PRRX1
ALDH4A1	-1.84	HOXA9, IRX3, IRX5
ALOX5	-1.84	HOXA9, IRX5
AMD1	-2.06	EMX2, HOXA5, HOXA9, HOXB5, PRRX1
ANGPT2	-1.55	HOXA9, IRX5
ANGPTL4	-2.12	HOXA9, IRX3
ANXA1	-1.64	HOXA5, HOXA9, HOXC6, IRX3
APOLD1	-5.98	HOXA5, HOXA9
AQP9	-2.94	HOXA9
ARID5B	-1.9	HOXA5, HOXA9, HOXC6
ARIH1	-1.58	EMX2, HOXA9, IRX3, IRX5
ARPC5L	-1.62	IRX3, IRX5, PRRX1
ARRDC4	-1.5	HOXA9, HOXB5, IRX3
ASPH	-1.63	EMX2, HOXA9, HOXB5, HOXC6, IRX3
ATF3	-8.87	HOXC6
ATP1B3	-1.63	HOXA9, HOXC6, IRX3
B3GNT5	-1.94	HOXC6, IRX5
BAG3	-3.51	HOXA9
BAMBI	-1.53	HOXC6, IRX3
BAZ1A	-1.66	HOXA9, HOXB5, HOXC6, IRX5
BCL3	-2.58	HOXC6, IRX5
BHLHB2	-4.1	IRX5
BIRC3	-1.57	HOXB5, IRX3
C13orf15	-1.76	HOXA5, HOXA9
C13orf18	-1.56	HOXA5, HOXA9, IRX5
C1orf108	-1.86	HOXA9
C1orf55	-1.73	HOXB5, IRX3
C21orf63	-1.88	IRX5
C6orf166	-1.55	HOXA5, HOXA9
C6orf32	-2.09	HOXA5, HOXA9, HOXB5, HOXC6
C8orf4	-1.81	HOXC6
CASP4	-1.51	HOXA5, HOXB5
CCNL1	-3.15	HOXA5, HOXA9, HOXC6, IRX3
CCRN4L	-3.42	HOXA9, IRX5
CD151	-1.55	HOXA9, IRX5
CD44	-2.06	EMX2, HOXA5, HOXA9, HOXB5, HOXC6, PRRX1
CD59	-1.82	EMX2, HOXA9, IRX5, PRRX1
CD69	-2.46	HOXA9, HOXB5
CD83	-2.79	HOXA9
CD93	-2.21	HOXA9
CD97	-1.9	HOXA9, HOXC6
CDKN1A	-10.07	HOXA9, IRX3, IRX5
CEACAM1	-2.41	HOXA5
CES1	-2.64	HOXA9
CFH	-1.59	HOXA5, HOXA9, HOXC6, IRX3, IRX5

CGGBP1	-1.62	HOXA9
CH25H	-5.57	IRX3
CHD1	-1.91	EMX2, HOXA5, HOXA9, HOXB5, HOXC6
CHD7	-1.58	EMX2, HOXA5, HOXA9, HOXB5, HOXC6, IRX3, IRX5, PRRX1
CHIC2	-1.79	HOXC6
CHST3	-1.66	HOXA9, HOXB5
CHSY1	-2.04	HOXA9, HOXB5, HOXC6, IRX3, IRX5
CKMT2	-1.52	HOXA9
CLCF1	-1.69	HOXA9, HOXC6, IRX5
CNKSR3	-2.16	HOXA5, HOXA9, HOXB5, IRX5
COQ10B	-1.84	HOXA9, HOXB5, IRX5
CRY1	-1.84	HOXA5, HOXA9, HOXC6, IRX3, IRX5, PRRX1
CRYAB	-1.87	IRX5
CSF3	-2.05	HOXA5, HOXC6
CSF3R	-3.68	HOXC6
CSNK1D	-1.75	HOXC6
CTSL1	-1.99	HOXA5, HOXA9, HOXC6
CXCL2	-8.36	IRX5
CXCR4	-2.27	HOXA9
DCUN1D3	-2.22	HOXA9
DDX21	-2.92	HOXA5, HOXA9, HOXC6, IRX3, IRX5, PRRX1
DNAJA1	-2.57	HOXA5, HOXA9, IRX5
DNAJA4	-1.51	HOXA9, HOXC6, IRX5
DPH3	-1.54	IRX5
DUSP1	-8.6	HOXA9
DUSP14	-1.86	HOXA9
DYRK3	-1.55	HOXA9, HOXB5, IRX3, IRX5
EBI2	-3.13	HOXA5
EFNB2	-1.95	HOXA5, HOXA9, HOXB5, IRX5
EGFL6	-5.68	HOXA9, HOXC6, IRX5
EGR2	-3.16	HOXA9
EGR3	-2.46	HOXA5, HOXA9
EHD1	-1.53	HOXB5, HOXC6
EIF4A3	-1.53	HOXA9, IRX3, PRRX1
EIF4G2	-1.55	EMX2, HOXA5, HOXA9, HOXB5, HOXC6, IRX3, IRX5
ELF1	-1.52	HOXA5, HOXA9, HOXB5, IRX5, PRRX1
EMP1	-2.49	HOXA9, HOXB5, IRX3, IRX5
ENDOGL1	-3.36	HOXA9, IRX5
ERRFI1	-2.92	HOXA9, PRRX1
ETS1	-1.61	HOXB5, IRX5
ETS2	-3.06	HOXA5, HOXA9, HOXC6, PRRX1
FAM107A	-2.19	HOXA5, HOXA9, HOXB5, IRX5
FAM53C	-1.61	HOXA5, HOXA9
FCGR3B	-1.72	HOXA5
FCN1	-2.12	HOXA9
FEM1C	-1.63	EMX2, HOXA5, HOXA9, HOXC6
FGR	-1.71	HOXA5, HOXA9, HOXC6, IRX5
FILIP1L	-2.26	HOXA5, HOXA9, HOXC6, IRX5, PRRX1
FOSL1	-2.59	HOXA9
FOSL2	-1.6	HOXA5
FPR1	-3.56	HOXA9
FPR2	-1.77	EMX2, HOXA9, IRX5, PRRX1
GADD45A	-2.12	HOXA9, HOXB5
GBP1	-1.59	EMX2, HOXB5, IRX5
GCA	-1.78	HOXA9, IRX5
GCH1	-1.64	HOXA9, IRX5
GEM	-2.55	HOXB5, HOXC6, IRX5
GJA1	-2.45	EMX2
GLRX	-1.52	HOXB5

GPR4	-2.27	HOXB5
GPR56	-2.11	HOXA9, IRX3, IRX5
GSPT1	-1.65	HOXA5, HOXA9, IRX3
GTF2B	-1.59	HOXA5, IRX5
GTPBP4	-1.52	HOXA9, IRX5
HAS1	-2.8	IRX5
HBB	-1.63	HOXA9
HBD	-1.6	HOXA9
HBEGF	-4.26	HOXC6
HBG2	-2.19	HOXA9, HOXB5, IRX3, IRX5
HIF1A	-2.1	HOXA5, HOXA9, HOXB5, IRX3
HIPK3	-1.51	HOXA9, HOXB5, HOXC6, IRX5
HIST1H2AC	-1.82	HOXA5
HIST2H2AA3	-1.63	IRX3
HLX	-1.61	HOXA5
HMOX1	-5.21	HOXA9
HNRNPAB	-1.6	HOXA9
HSD11B1	-2.21	HOXA9, IRX3, PRRX1
HSPA1B	-3.02	HOXC6
HSPA8	-1.52	HOXC6
HSPB8	-2.26	HOXA9, HOXC6, IRX5
HSPH1	-2.86	HOXA9, HOXC6
ICAM2	-1.53	HOXA9
IER3	-5.22	HOXC6, IRX5
IER5	-1.94	HOXA9
IFI16	-1.84	HOXA5, HOXA9, HOXC6, IRX5
IFIT3	-1.55	HOXA9, IRX5
IFRD1	-1.64	EMX2, HOXA5, HOXA9, HOXC6, IRX3, IRX5
IL18RAP	-1.88	HOXA9, HOXC6
IL1B	-4.76	EMX2, HOXA5, HOXA9, HOXB5, IRX5
IL1R2	-1.93	IRX5
IL1RL1	-2.06	HOXA9, HOXB5, HOXC6
IL6	-25.68	EMX2
IL8	-19.53	HOXA5, HOXB5
IL8RB	-1.69	HOXA5, IRX5
INPP1	-1.5	HOXA5, HOXA9
INSIG1	-2.3	HOXA5, IRX5
IRAK2	-1.53	HOXC6
IRF7	-2.03	EMX2
ISG20	-1.9	HOXA9
ISG20L1	-1.99	EMX2, IRX3
ITGA5	-1.76	HOXA9
ITIH5	-1.6	HOXC6, IRX3
IVNS1ABP	-1.66	HOXA5, HOXA9, HOXB5, HOXC6, IRX5
JMJD1C	-1.8	HOXA9, HOXB5, HOXC6, IRX5
KBTBD2	-1.6	HOXA9, HOXB5
KCNJ2	-1.81	IRX5
KIF5B	-1.75	HOXA5, HOXA9, IRX5
KIAA0247	-1.51	HOXB5, HOXC6
KIAA1434	-1.5	HOXA5, HOXA9, HOXB5, HOXC6, IRX5
KIAA1754	-2.06	HOXA5
KLF10	-3.26	HOXB5, IRX3
KLF4	-5.03	HOXA5
KLF9	-1.59	IRX3, IRX5
LBP	-2.41	HOXA9
LDLR	-10.41	HOXA9
LILRA3	-1.65	EMX2, HOXB5
LITAF	-1.63	HOXA9
LMCD1	-1.62	HOXC6, IRX3, IRX5

LOH3CR2A	-1.57	HOXC6, IRX3, IRX5
LONRF3	-1.51	EMX2, HOXA5
LRG1	-2.04	HOXA9
LRRC32	-1.82	HOXA5, IRX5
MAFF	-1.93	HOXA9, IRX3, IRX5
MALL	-1.54	HOXA5
MAP1B	-1.62	HOXA5, HOXA9, HOXC6
MAP3K8	-3.7	HOXA5, HOXA9
MAT2A	-3.33	HOXA5, PRRX1
MCL1	-5.31	HOXA9
METRNL	-1.56	HOXA9
MGC42367	-1.88	HOXA5, HOXA9, HOXC6, IRX3
MGC4677	-2.17	IRX3
MGST1	-1.52	EMX2, HOXA9, HOXB5, IRX3, IRX5
MLKL	-1.73	HOXA9
MMP19	-1.83	HOXA5, IRX5
MNDA	-2.06	HOXA5, HOXB5
MRAP	-1.75	IRX5
MSC	-2.32	HOXA9, IRX5
MT1M	-3.67	HOXA5
MTHFD2	-2.03	HOXA5
MXRA7	-1.52	HOXA5, HOXA9
MYC	-7.23	HOXA9
MYOC	-1.8	HOXA9
NAMPT	-5.48	HOXA9, IRX3
NCOA7	-1.74	EMX2, HOXA5, HOXA9, HOXC6, IRX3, IRX5, PRRX1
NDEL1	-1.81	HOXA9, HOXB5, IRX5
NEDD9	-1.59	HOXA9, IRX5
NFATC1	-2.28	HOXA9
NFE2L2	-1.5	HOXA5, HOXA9, HOXC6, IRX3
NFKBIZ	-5.96	HOXA9, HOXB5, IRX3
NIP7	-1.72	HOXA9
NLRP3	-1.6	HOXA9, IRX5
NNMT	-2.04	HOXA9, IRX5, PRRX1
NOP58	-1.5	HOXA9, HOXB5, HOXC6, IRX3, IRX5
NP	-6.05	HOXC6, IRX5
NQO1	-2.8	HOXC6
NR4A2	-5.17	HOXA5, HOXA9, IRX5
NR4A3	-2.03	HOXA9
NUFIP2	-1.81	HOXA9, HOXC6
NXT1	-2.05	IRX5
OBFC2A	-2.26	HOXA9, IRX3
OLR1	-2.09	HOXA5, HOXC6, IRX5
OSMR	-1.97	HOXA9
PANX1	-1.66	IRX5
PCDH17	-1.6	HOXA9, HOXB5, HOXC6
PDE4B	-1.56	EMX2, HOXA5, HOXA9, HOXB5, HOXC6, IRX3, IRX5, PRRX1
PDLIM1	-1.68	HOXA5
PELI1	-2.23	HOXA9, HOXC6
PELO	-1.52	HOXA5, HOXA9, HOXB5, HOXC6, IRX3, IRX5
PHLDA1	-5.05	HOXA5, IRX3
PI3	-1.57	HOXA5, PRRX1
PIM1	-4.34	HOXA9, HOXC6, IRX3
PLAU	-2.81	IRX5
PLAUR	-3.86	EMX2
PLOD2	-1.64	EMX2, HOXB5
PLSCR1	-1.74	HOXB5, HOXC6, PRRX1
PMAIP1	-1.6	HOXA5
PMEPA1	-1.71	HOXA9, IRX5

POLR1C	-1.68	PRRX1
PPA1	-1.54	HOXC6
PPAP2A	-1.6	EMX2, HOXA9, HOXB5, HOXC6, IRX5
PPP1R15A	-4.11	PRRX1
PPP2R1B	-1.86	HOXA9
PPRC1	-2.92	HOXA9, IRX5
PPTC7	-1.52	IRX5
PRDM1	-1.8	IRX3, IRX5
PRIC285	-2.42	HOXA5
PROK2	-4.66	HOXA9, IRX3, IRX5
PSCD1	-1.69	HOXA9, HOXC6, IRX5
PTGER4	-2.02	HOXA5
PTGS2	-9.44	EMX2, HOXA9, HOXC6, IRX5
PTPN1	-1.56	HOXA5, HOXA9
PTPRE	-1.77	EMX2, HOXA5, HOXA9, IRX5
PUS1	-1.57	HOXB5
RARA	-1.64	HOXA9
RASIP1	-1.76	HOXA9
RCAN1	-6.04	HOXA5, HOXA9, HOXB5, HOXC6, IRX3, IRX5
RFX2	-1.55	HOXC6, IRX5
RGS1	-1.93	HOXA9, HOXC6, IRX5
RHOB	-1.92	HOXA9
RND3	-3.83	HOXA9, HOXC6, IRX5
RRP12	-2.1	IRX3, IRX5, PRRX1
RYBP	-1.53	IRX5
S100A12	-2.85	HOXA9
S100A8	-10.87	HOXA9, IRX5
S100A9	-6.07	IRX5
SAT1	-1.85	HOXA9
SBNO2	-1.64	HOXA5
SCHIP1	-1.77	HOXA9
SDS	-1.67	EMX2
SEC14L1	-1.61	EMX2, HOXA5, IRX5
SELL	-2.13	HOXA5, HOXA9
SELP	-1.75	HOXA5, HOXC6, IRX5
SERPINA1	-1.97	IRX5
SERPINA3	-1.83	HOXA5, IRX3, PRRX1
SERPINB1	-1.72	HOXA9, IRX5
SERPINB8	-1.56	HOXA5, PRRX1
SERPINE1	-5.62	HOXA9, HOXB5, IRX5
SGK	-4.78	HOXA5, HOXA9, HOXB5
SLC11A1	-2.13	IRX5
SLC16A3	-1.88	HOXA5
SLC20A1	-2.8	HOXA5, HOXA9, HOXB5
SLC25A25	-6.78	HOXA9, HOXB5
SLC25A37	-1.77	HOXA5, HOXA9, HOXB5
SLC25A44	-1.98	HOXA5
SLC2A14	-2.89	HOXA5, HOXA9, PRRX1
SLC2A3	-8.11	HOXA5, HOXA9, IRX3
SLC31A2	-1.66	EMX2, HOXA5, HOXA9, HOXC6, IRX3, IRX5, PRRX1
SLC38A2	-1.68	EMX2, HOXA5, HOXA9, HOXC6, IRX5, PRRX1
SLC39A14	-2.24	HOXA9, IRX5, PRRX1
SLCO2A1	-1.62	HOXA9
SLCO4A1	-1.55	HOXA9
SMAD7	-1.53	HOXA9
SNCG	-2.01	HOXA9
SNF1LK	-3.24	HOXC6
SORL1	-1.85	HOXA5, HOXA9, HOXB5, HOXC6, IRX5
SOX7	-3.14	HOXA5, HOXA9, IRX3, IRX5

SPHK1	-1.77	HOXA5, IRX5
SPRY4	-1.78	HOXA5, HOXA9, HOXC6, IRX5
SPSB1	-1.9	HOXA9
SRPX2	-1.52	HOXA9, IRX5
STARD13	-1.56	EMX2, HOXA5, HOXA9, HOXB5, HOXC6, IRX3, IRX5
STC1	-4.14	HOXA5, HOXB5
STOM	-1.69	EMX2, HOXA5, HOXA9, IRX3, IRX5
TAP1	-1.86	HOXA5, HOXA9, IRX3, IRX5
TEAD4	-1.98	HOXA9, HOXB5, HOXC6, IRX5
TFPI2	-1.52	HOXA9, IRX5
TFRC	-1.99	HOXA5, HOXB5, HOXC6
THBD	-2.14	IRX5
THBS1	-3.84	HOXA9, IRX5
TICAM1	-1.93	HOXA9
TIPARP	-4.16	HOXA5, HOXA9
TM4SF1	-2.99	HOXA9, IRX3
TMED5	-1.51	HOXA9
TMEM154	-1.72	HOXA5, HOXA9, IRX5
TMEM173	-1.69	HOXC6
TMEM2	-3.65	HOXA5, HOXA9, IRX3, IRX5, PRRX1
TMEM49	-1.88	EMX2, HOXA9, HOXB5, IRX5
TMEM71	-1.66	HOXA9, IRX3
TNFAIP1	-1.62	IRX5
TNFAIP3	-2.12	HOXA5, HOXA9, HOXC6
TNFRSF10B	-1.97	HOXA9
TNMD	-2.6	HOXA9
TPST2	-1.51	HOXA9, HOXB5, HOXC6, PRRX1
TRAF3IP2	-1.59	EMX2, HOXA9, HOXC6
TRIB1	-6	IRX5
TSC22D1	-1.8	HOXC6
TSC22D2	-2.79	HOXA9, HOXC6, IRX5
TUBB2C	-1.7	HOXA9
TUBB3	-1.89	HOXA9, IRX5
TUBB4Q	-1.54	HOXC6, IRX5
TUBB6	-1.5	HOXA5, HOXA9, HOXC6
TXNRD1	-1.72	EMX2, HOXA5, HOXA9, HOXB5, HOXC6, IRX5
UAP1	-2.06	HOXC6, IRX3, PRRX1
UBAP1	-1.53	EMX2, HOXA9, HOXB5, IRX3
UCHL1	-2.23	HOXA5, HOXA9
UGCG	-2.85	EMX2, HOXA9
UPP1	-2.18	HOXA9, PRRX1
VEGFA	-1.56	HOXB5
VIL2	-1.71	HOXA9, HOXC6
VNN2	-3.73	IRX3, IRX5
VNN3	-1.56	EMX2, IRX5
WARS	-1.52	HOXA9
WDR52	-1.57	EMX2, HOXA5, HOXA9, HOXB5, HOXC6, IRX5
WTAP	-1.6	HOXA9, HOXC6
YRDC	-1.85	HOXA5, HOXB5, IRX3
ZC3H12A	-2.62	HOXA9, IRX5
ZNF295	-1.5	PRRX1
ZNF331	-1.75	HOXA9, IRX5
ZSWIM4	-1.64	HOXA9, HOXC6
