# Supplementary material for: Switch from Stress Response to Homeobox Transcription Factors in Adipose Tissue After Profound Fat Loss
Source: PLoS One. 2010 Jun 9;5(6):e11033. doi: 10.1371/journal.pone.0011033 (PMC2882947; doi:10.1371/journal.pone.0011033)
Supplement: Table S7 — Up-regulated genes in adipose tissue after bariatric surgery that contain one or more binding sites for one or more homeobox transcription factors. (0.01 MB PDF) [file pone.0011033.s007.pdf]

TABLE S7 Up-regulated genes in adipose tissue after bariatric surgery that contain one or more binding sites for one or more homeobox transcription factors

Target gene	Post/Pre	Potential regulators
ABCA10	1.59	HOXA9, HOXC6, IRX5
ADD3	1.63	EMX2, HOXA5, HOXA9, HOXC6, IRX5, PRRX1
AGTR1	1.89	HOXA9, IRX3, IRX5, IRX5
APOC1	3.21	HOXA5
BHLHB5	1.64	IRX5
C10orf6	1.65	HOXA5, HOXA9, HOXB5, HOXC6, IRX5, PRRX1
C14orf106	1.57	HOXA5, HOXA9, HOXB5, HOXC6, IRX5
C14orf28	1.56	HOXA5, HOXA9, HOXC6
C17orf58	2.75	HOXA5, IRX5
C1orf186	1.54	HOXA9
C20orf177	1.77	IRX3
C5orf13	1.8	EMX2, HOXA5, HOXA9, HOXB5, HOXC6, IRX5
C6	2.97	HOXA9, IRX3, IRX5
C6orf192	1.58	HOXA5, IRX3
CA12	1.95	HOXB5, IRX5
CCNB1IP1	1.55	HOXA5, HOXA9, IRX3, IRX5
CDKN1B	1.89	HOXA5
CDKN2C	1.71	HOXA5
CETP	2.34	HOXA9
CIRBP	1.55	IRX5
CLEC3B	1.58	IRX3, IRX5
COL1A2	4.22	HOXA5, IRX3
COL3A1	2.11	HOXA9, HOXC6, IRX5
COL5A1	1.62	HOXB5, IRX3, IRX5
COL6A3	1.67	HOXB5, HOXC6, IRX3, PRRX1
CPA3	1.76	PRRX1
CPXM1	2.09	HOXA5, IRX5
CPZ	1.72	HOXA5
CTHRC1	1.52	HOXA5
CTSK	1.99	HOXB5, HOXC6, IRX3
DCLK1	1.98	HOXA5, HOXA9, HOXB5, HOXC6, IRX5
DENND2A	1.6	HOXA5
DFNA5	1.77	HOXA9, HOXC6, IRX3
DPP4	1.65	IRX3, IRX5, PRRX1
DPYSL3	1.52	IRX3
DYRK2	1.51	HOXA9, HOXB5, HOXC6, IRX3, IRX5
FAM14A	1.6	HOXA9, HOXB5, HOXC6
FAM84B	1.69	HOXA9, IRX3
FNDC1	3.6	HOXA5, HOXA9, HOXB5, HOXC6, IRX3
FRMD6	1.77	HOXA9, HOXB5, HOXC6, IRX5
GALNTL1	1.53	EMX2, HOXA9, HOXB5, HOXC6, IRX5
GCHFR	1.84	HOXA5, IRX5
GIMAP8	1.65	HOXA5, IRX5
GPD1L	1.65	IRX5
GPNMB	1.78	HOXA9, HOXC6
GSDML	1.76	HOXA5, PRRX1
HNRPDL	1.55	HOXA5, HOXA9, IRX5
HOXA5	2.38	IRX5
HOXA9	2.29	HOXA9, IRX3, PRRX1
HOXB5	1.52	HOXC6
HOXC6	1.86	EMX2, HOXA9
IGFBP5	1.54	HOXA5
IL11RA	1.62	HOXB5

IRX5	1.5	HOXA9
ITGA11	1.52	IRX3
KIT	1.78	EMX2, HOXA5, HOXA9, HOXB5, HOXC6
KIAA1712	1.58	EMX2, HOXA5, IRX5
LGALS3	1.52	EMX2, HOXA5, HOXA9
LMO3	1.84	HOXA5, HOXA9, HOXC6, IRX3, IRX5
LPAR1	1.59	HOXA9, HOXC6, IRX3
LRRC17	2.78	HOXA5, HOXA9, HOXB5, IRX5, PRRX1
MARCKS	1.71	HOXA9, HOXC6
MDK	1.62	IRX5
NENF	1.52	HOXA5, HOXA9, IRX5
NKTR	1.61	HOXA5, HOXA9, HOXB5, HOXC6, IRX3
NOPE	1.97	HOXB5
NR1H3	1.55	HOXA5, HOXB5
OGT	1.5	HOXA5, HOXA9, IRX3
OLFML3	2.05	IRX3
PCDH18	2.33	HOXA9, IRX5
PCK2	1.69	IRX5
PCOLCE	2.1	HOXB5
PDGFD	1.5	HOXA5, HOXA9
PDGFRL	1.88	HOXA5
PEX11B	1.5	HOXA9
PKD1L2	2	HOXA5, HOXA9, IRX5, PRRX1
PMM1	1.64	HOXA9, IRX3, IRX5, PRRX1
PRICKLE1	1.65	HOXA9, HOXB5, HOXC6
PRRX1	1.76	HOXA5, IRX5
PTGES	1.51	HOXB5
PTGIS	1.65	HOXA9, HOXC6, IRX5
RAB23	1.53	EMX2, HOXA9
RBM33	1.72	HOXA9, HOXB5, HOXC6, IRX3, IRX5, PRRX1
RCOR3	1.7	EMX2, HOXA5, HOXC6, PRRX1
RPL13	1.54	IRX5
RPL13A	1.5	EMX2, HOXA9, HOXC6
RPL14	1.7	HOXA9, HOXC6, IRX3, IRX5
RPL21	1.51	HOXA5
RPL9	1.52	HOXC6, IRX3
RPS27	1.53	EMX2
RUNX1T1	1.53	HOXA5, HOXA9, HOXB5, HOXC6, IRX5
SF3A2	1.7	IRX5
SH3PXD2A	1.55	EMX2, HOXA5, HOXA9, HOXC6, IRX5
SLC40A1	1.78	EMX2, HOXA9, HOXC6, IRX3
SREBF1	1.77	IRX3
ST3GAL5	1.75	HOXA5, HOXA9, IRX3, IRX5
STAG3L2	1.77	HOXB5, HOXC6, IRX3, IRX5
THRA	1.82	EMX2, HOXA5, HOXA9, IRX3, IRX5
TM7SF2	1.69	HOXA9, HOXB5, IRX5
TNFSF10	1.5	IRX3
TRAPPC2L	1.51	HOXA9
TUBB	1.82	HOXA9
VAT1	1.62	HOXA9, HOXC6, IRX5
WDR33	1.53	HOXA5, HOXA9, HOXB5, HOXC6, IRX3
ZADH2	1.7	IRX5
ZKSCAN4	1.54	HOXA9
ZNF395	1.55	HOXA9, HOXC6
ZNF564	1.53	IRX5
ZNF573	1.59	HOXA5, HOXA9, HOXB5, PRRX1
ZNF589	1.51	HOXA9, IRX5
