# Supplementary material for: Switch from Stress Response to Homeobox Transcription Factors in Adipose Tissue After Profound Fat Loss
Source: PLoS One. 2010 Jun 9;5(6):e11033. doi: 10.1371/journal.pone.0011033 (PMC2882947; doi:10.1371/journal.pone.0011033)
Supplement: Table S8 — PANTHER categories enriched with differentially expressed genes that contain one or more binding sites for one or more homeobox transcription factors. (0.02 MB PDF) [file pone.0011033.s008.pdf]

TABLE S8 PANTHER categories enriched with differentially expressed genes that contain one or more binding sites for one or more homeobox transcription factors

A. Down-regulated genes in adipose tissue after bariatric surgery

PANTHER Molecular Function	Potential targets of EMX2, HOXA5, HOXA9, HOXB5, HOXC6, IRX3, IRX5 and/or PRRX1	Count
**Molecular function unclassified**	ADFP, BCL3, BYSL, CD44, CKS2, NQO1, HBB, IFRD1, TM4SF1, MGST1, MT1A, MT1E, MT1G, MT1M, MT2A, ODC1, PMAIP1, SRGN, SEC14L1, TNFAIP1, BTG2, CGGBP1, IER3, GPRC5A, NOLC1, CD83, WTAP, AKAP12, C6orf32, KIAA0247, CDC42EP2, IVNS1ABP, TRAF3IP2, MTHFD2, FAM107A, FILIP1L, PHLDA1, RRP12, CDC42EP4, TMEM2, PANX1, FJX1, BAMBI, KBTBD2, EGFL6, CHIC2, SRPX2, C13orf15, LOH3CR2A, SCHIP1, LMCD1, UBAP1, IER5, FAM53C, C4orf18, TNFRSF12A, ERRFI1, SLC38A2, DDX21, RASIP1, WDR52, MRAP, C8orf4, PNO1, FEM1C, PMEPA1, PELI1, NUFIP2, C21orf63, C12orf44, NFKBIZ, AXUD1, ZSWIM4, C1orf108, YRDC, LONRF3, GRRP1, SPSB1, C13orf18, NDEL1, APOLD1, TMEM49, CRISPLD2, C13orf33, C17orf91, KIAA1754, MIDN, C19orf22, MYADM, ARRDC4, TMEM88, NCRNA00152, NLRP3, DCUN1D3, SPATA2L, NCOA7, TMEM71, SRXN1, CMTM2, C1orf55, C19orf59, TMEM154, METRNL, DPH3, TMEM173, C2orf55, C2CD4B, MXRA7	**108**
**Transcription factor**	JMJD3	**67**
Zinc finger transcription factor	PRDM1, KLF9, KLF6, KLF10, KLF4, KLF2, ZNF295, JMJD1C	
HMG box transcription factor	SOX17, SOX7	
KRAB box transcription factor	EGR1, EGR2, EGR3, VNN3	
Basic helix-loop-helix transcription factor	AHR, HIF1A, MXD1, MYC, BHLHB2, MSC, HES4	
Nuclear hormone receptor	NR4A1, NR4A2, RARA, NR4A3	
Transcription cofactor	ELL, ARID5A, PPRC1, RYBP, CCNL1, ARID5B	
Other transcription factor	CEBPD, ELF1, ETS1, ETS2, FOXC1, FOS, FOSB, FOSL2, IFI16, IRF1, IRF7, JUN, JUNB, JUND, SMAD7, MNDA, NFATC1, NFE2, NFE2L2, NFIL3, RFX2, TEAD4, FOSL1, TSC22D1, LITAF, TSC22D2, SPRY1, MAFF, SERTAD1, DNAJA4, SPRY4	
**Nucleic acid binding**	ATF3, CEBPB, CEBPB, CEBPD, EGR1, EGR2, EGR3, FOXC1, FOS, FOSB, FOSL2, HIF1A, IRF1, IRF7, JUN, JUNB, JUND, MYC, NFE2, NFE2L2, NOL1, RARA, FOSL1, BHLHB2, MSC, MAFF, CCRN4L, TIPARP, DNAJA4, HES4, SOX17, OBFC2A, ZC3H12A, SOX7	**63**
Helicase	SBNO2, PRIC285	
RNA helicase	DDX3X, DDX5, DDX21, EIF4A3	
DNA helicase	CHD1, CHD7	
Nuclease	SBNO2, PELO	
Exoribonuclease	ISG20, ISG20L1	
Histone	HIST1H2AC, HIST2H2AA3	
Other DNA-binding protein	PRDM1, HLX	
Ribonucleoprotein	HNRNPAB, NOP58	
Other RNA-binding protein	ZFP36, RBM15	

Translation factor		
Translation initiation factor	EIF4G2, EIF4A3	
Translation release factor	GSPT1, PELO	
**Receptor**	LRRC32, MYOC, SELP, SLC20A1, HYAL2, LRG1	**41**
G-protein coupled receptor	C5AR1, CD97, EBI2, FPR1, FPR2, DARC, GPR4, FFAR2, IL8RB, PTGER4, CXCR4, GPR56, GPR109A	
Other receptor	AHR, CD69, LDLR, OLR1, TNFRSF11B, PLAUR, PVRL2, SORL1, TFRC, THBD, TNFRSF4, CD93	
Serine/threonine protein kinase receptor	IRAK2, PIM1	
Cytokine receptor		
Interleukin receptor	IL1R2, IL18RAP, IL1RL1	
Tumor necrosis factor receptor	TNFRSF10D, TNFRSF10B	
Other cytokine receptor	CX3CL1, OSMR	
**Signaling molecule**	ICAM2, ANGPTL4	**37**
Cytokine		
Interleukin	IL1B, IL6, OSM, CLCF1	
Chemokine	CXCL2, IL8, CCL2, CCL3, CCL3L1, CCL8, CCL4L1, CCL3L3	
Other signaling molecule	ANGPT2, BMP2, RCAN1, ETS1, ETS2, MCL1, SNCG, THBS1, SPRY1, PROK2, SPRY4	
Growth factor	HBEGF, CYR61, VEGFA	
Membrane-bound signaling molecule	CD59, CD151, EFNB2, NOTCH3, TMED5, TNMD	
Peptide hormone	ADM, STC1, GNL3	
**Select regulatory molecule**	FSTL3	**35**
G-protein		
Small GTPase	RHOB, RND3, GEM, ARL4A, GTPBP4, RND1, RASD1, ARL5B	
G-protein modulator		
Other G-protein modulator	RGS1, RGS2, EHD1, STARD13	
Kinase modulator	ANGPT2, CCNL1, CNKSR3	
Kinase inhibitor	CDKN1A, DUSP1, DUSP5, DUSP14	
Other enzyme regulator	ANGPT2, THBS1	
Protease inhibitor	BIRC3, CASP4	
Serine protease inhibitor	SERPINA3, SERPINB1, SERPINE1, SERPINA1, PI3, SERPINB8, TFPI2, ITIH5	
**Transferase**	MAP3K8, MAP2K3, STC1	**22**
Glycosyltransferase	HAS1, HAS2, UGCG, B4GALT5, CHSY1, B3GNT5	
Acetyltransferase	SAT1, BAZ1A	
Methyltransferase	NNMT, NOL1	
Nucleotidyltransferase	MAT2A, UAP1	
Phosphorylase	NP, UPP1	
Other transferase	ALAS2, TGM2, TPST2, CHST3	

**Oxidoreductase**	NOS3	**18**
Dehydrogenase	HSD11B1, RDH5, ALDH4A1, ALDH1A2	
Oxygenase	ALOX5, HMOX1, PLOD2, PTGS2	
Reductase	AKR1C2, HSD11B1, RDH5, TXNRD1	
Hydroxylase	ASPH, CH25H	
Other oxidoreductase	GLRX, SOD2	
**Kinase**	ITPKC	**18**
Protein kinase	MAP3K8, IRAK2, PIM1, MAP2K3, TRIB1, MLKL	
Non-receptor serine/threonine protein kinase	CDK2, CSNK1D, SGK, DYRK3, HIPK3, SNF1LK	
Non-receptor tyrosine protein kinase	ABL2, FGR, DYRK3	
**Transporter**	SORL1	**15**
ATP-binding cassette (ABC) transporter	TAP1, ABCC3	
Carbohydrate transporter	SLC2A3, SLC2A14	
Cation transporter	ATP1B3, SLC31A2	
Other transporter	AQP9, SLC11A1, SLC20A1, SLCO2A1, SLC16A3, SLC39A14, SLCO4A1	
**Defense/immunity protein**	FCGR3B, NOTCH3, LILRB3, LILRA3, LILRA5	**14**
Immunoglobulin receptor family member	FCGR3B, LILRB3, LILRA3, LILRA5	
Antibacterial response protein	FCN1, LBP	
Other defense and immunity protein	CD69, PVRL2	
**Miscellaneous function**	IFIT3	**14**
Structural protein	CRYAB, LMNA	
Other miscellaneous function protein	ADM, GADD45A, INSIG1, GADD45B, SNCG, SOCS2, SOCS3, GADD45G, PPP1R15A, NXT1, NIP7	
**Transfer/carrier protein**	ANXA1, LBP, TICAM1	**11**
Mitochondrial carrier protein	SLC25A44, SLC25A37, SLC25A25	
Other transfer/carrier protein	HBA2, HBD, HBG1, HBG2, PLSCR1	
**Cytoskeletal protein**		**10**
Microtubule family cytoskeletal protein		
Tubulin	TUBB2A, TUBB3, TUBB2C, TUBB4Q, TUBB6	
Other cytoskeletal protein	EMP1, STOM, NEDD9, SNCG	
**Phosphatase**		**10**
Protein phosphatase	DUSP1, DUSP5, PPP2R1B, PTPN1, PTPRE, DUSP14, PPTC7	
Other phosphatase	INPP1, PPAP2A	
**Hydrolase**	TNFAIP3, ENDOGL1	**10**
Other hydrolase	GCH1, VNN2, PADI4, C6orf166	
Phosphodiesterase	PDE4B, GPCPD1	

**Chaperone**	BAG3	**9**
Hsp 70 family chaperone	HSPA1B, HSPA8, HSPH1	
Other chaperones	DNAJA1, DNAJB1, HSPB8, ZNF331, GRPEL1	
**Protease**		**8**
Metalloprotease	MMP19, ADAM19, ADAMTS1, ADAMTS9, MMP25	
Cysteine protease	CASP4, CTSL1, UCHL1	
**Select calcium binding protein**	EHD1, GCA	**8**
Calmodulin related protein	NOS3, S100A8, S100A9, S100A12, S100P	
**Cell adhesion molecule**	CD151	**7**
Other cell adhesion molecule	ITGA5, SELL, SELP	
CAM family adhesion molecule	CEACAM1, ICAM2	
**Synthase and synthetase**	PTGS2	**7**
Synthase	ALAS2, HAS1, HAS2, NOS3, PUS1	
**Extracellular matrix**	LRRC32, ANGPTL4, LRG1	**6**
Other extracellular matrix	MMP19, THBS1, MMP25	
**Membrane traffic protein**	MALL, EHD1, CHMP1B	**3**

B. Up-regulated genes in adipose tissue after bariatric surgery

PANTHER Molecular Function	Potential targets of EMX2, HOXA5, HOXA9, HOXB5, HOXC6, IRX3, IRX5 and/or PRRX1	Count
**Molecular function unclassified**	C10orf6, C14orf106, C14orf28, C17orf58, C1orf186, C20orf177, C5orf13, CCNB1IP1, DENND2A, DFNA5, FAM14A, FNDC1, FRMD6, GCHFR, GPNMB, GSDML, KIAA1712, PEX11B, RBM33, SH3PXD2A, SLC40A1, TM7SF2, TRAPPC2L	**23**
**Nucleic acid binding**	BHLHB5, HOXA5, HOXB5, HOXC6, IRX5, NR1H3, ZNF307	**22**
Ribosomal protein	RPL13, RPL13A, RPL14, RPL21, RPL9, RPS27	
Other DNA-binding protein	HOXA9, PRRX1, RCOR3	
**Transcription factor**		**17**
Homeobox transcription factor	HOXA5, HOXA9, HOXB5, HOXC6, IRX5, PRRX1	
KRAB box transcription factor	ZNF307, ZNF564, ZNF573, ZNF589	
Basic helix-loop-helix transcription factor	BHLHB5, SREBF1	
Nuclear hormone receptor	NR1H3, THRA	
Transcription cofactor	RUNX1T1, ZNF395	
**Extracellular matrix**	LRRC17	**7**
Extracellular matrix structural protein	CLEC3B, COL1A2, COL3A1, COL5A1, COL6A3	

**Signaling molecule**	NENF	**7**
Other signaling molecule	LGALS3, MARCKS	
**Kinase**		**6**
Protein kinase	KIT, PDGFRL	
Non-receptor serine/threonine protein kinase	DCAMKL1, DYRK2	
Protein kinase receptor		
Tyrosine protein kinase receptor	KIT, PDGFRL	
**Select regulatory molecule**	FAM84B	**6**
Kinase modulator		
Kinase inhibitor	CDKN1B, CDKN2C	
**Protease**		**5**
Metalloprotease	CPA3, CPXM1, CPZ	
**Cytoskeletal protein**		**5**
Actin binding cytoskeletal protein	PRICKLE1	
Non-motor actin binding protein	ADD3, MARCKS	
**Miscellaneous function**	WDR33	**5**
Structural protein	MARCKS, PRICKLE1	
Other miscellaneous function protein	GIMAP8, IGFBP5	
**Oxidoreductase**	PTGIS	**4**
Dehydrogenase	GPD1L, VAT1, ZADH2	
Reductase	VAT1, ZADH2	
**Receptor**	LRRC17, OLFML3	**5**
G-protein coupled receptor	AGTR1, LPAR1	
**Cell adhesion molecule**		**3**
Other cell adhesion molecule	ITGA11, LGALS3	
**Transferase**		**3**
Glycosyltransferase	GALNTL1, OGT, ST3GAL5	
**Transfer/carrier protein**		**3**
Apolipoprotein	APOC1, APOE	
**Defense/immunity protein**		**2**
Complement component	C6, CTHRC1	
**Isomerase**	PTGES, PTGIS	**2**
