# Supplementary material for: Switch from Stress Response to Homeobox Transcription Factors in Adipose Tissue After Profound Fat Loss
Source: PLoS One. 2010 Jun 9;5(6):e11033. doi: 10.1371/journal.pone.0011033 (PMC2882947; doi:10.1371/journal.pone.0011033)
Supplement: Text S1 — Materials and methods (AB 1700 Expression Array system) (0.03 MB DOC) [file pone.0011033.s009.doc]

**TEXT S1**

**Materials and Methods (AB 1700 Expression Array system)**

A pilot microarray study of adipose tissue from 9 of the patients before and after surgery was performed using the Applied Biosystems (AB) 1700 Expression Array system. The AB 1700 system is based on chemiluminescence. Using the Applied Biosystems NanoAmpTM RT-IVT Labeling Kit, 540ng of total RNA from each sample was reversely transcribed, amplified and DIG-labeled (DIG-dUTP; Roche, Germany). The amount (20-80 μg) and quality of labelled cRNA were measured using both NanoDrop spectrophotometer and Agilent 2100 Bioanalyzer. Fifteen μg of the cRNA was hybridized to the Applied Biosystems Human Genome Survey Microarray V2.0 according to the manufacturer instructions. The AB human microarray V2.0 contains 32,878 probes representing 29,098 genes, together with about 1,000 control probes.

*Quality control and preprocessing of AB 1700 data*. The raw data were imported and analysed in the microarray analysis software J-Express 2.7 [1] ([www.molmine.com](http://www.molmine.com/)). All flagged spots and control spots were removed (Flags>5000.0, Probe_type != probe). Before compiled into an expression profile data matrix, all arrays were quantile normalized to be comparable. Genes with at most 30 % missing values were allowed in the final dataset. Missing values were replaced using the LSimpute_adaptive method [2]. The signal intensities in the dataset were log transformed (base 2). Correspondence Analysis was performed to obtain a global view of the data [3]. Both genes and samples can be plotted in the same space. The microarray data are projected onto a two-dimensional plane defined by the first and second principal components. The first principal component (along the x-axis) explains most of the total chi square, and the second principal component explains the second most of the total chi square. Samples that are close together in the plot are more similar than samples further apart. The plot showed both Pre_17 and Post_17 samples as outliers and suggested that Pre_11 and Post_11 had been mislabeled. Pre_11 and Post_11 were removed from the analysis. RNA from these samples was re-extracted for the main study. Pre_17 and Post_17 were kept in the analysis as no technical reason for these samples behaving differently could be identified.

**References**

1. Dysvik B, Jonassen I (2001) J-Express: exploring gene expression data using Java. Bioinformatics 17: 369-370.

2. Bo TH, Dysvik B, Jonassen I (2004) LSimpute: accurate estimation of missing values in microarray data with least squares methods. Nucleic Acids Res 32: e34.

3. Fellenberg K, Hauser NC, Brors B, Neutzner A, Hoheisel JD, et al. (2001) Correspondence analysis applied to microarray data. Proc Natl Acad Sci U S A 98: 10781-10786.
