# Supplementary figures and images for: Switch from Stress Response to Homeobox Transcription Factors in Adipose Tissue After Profound Fat Loss
Source: PLoS One. 2010 Jun 9;5(6):e11033. doi: 10.1371/journal.pone.0011033 (PMC2882947; doi:10.1371/journal.pone.0011033)

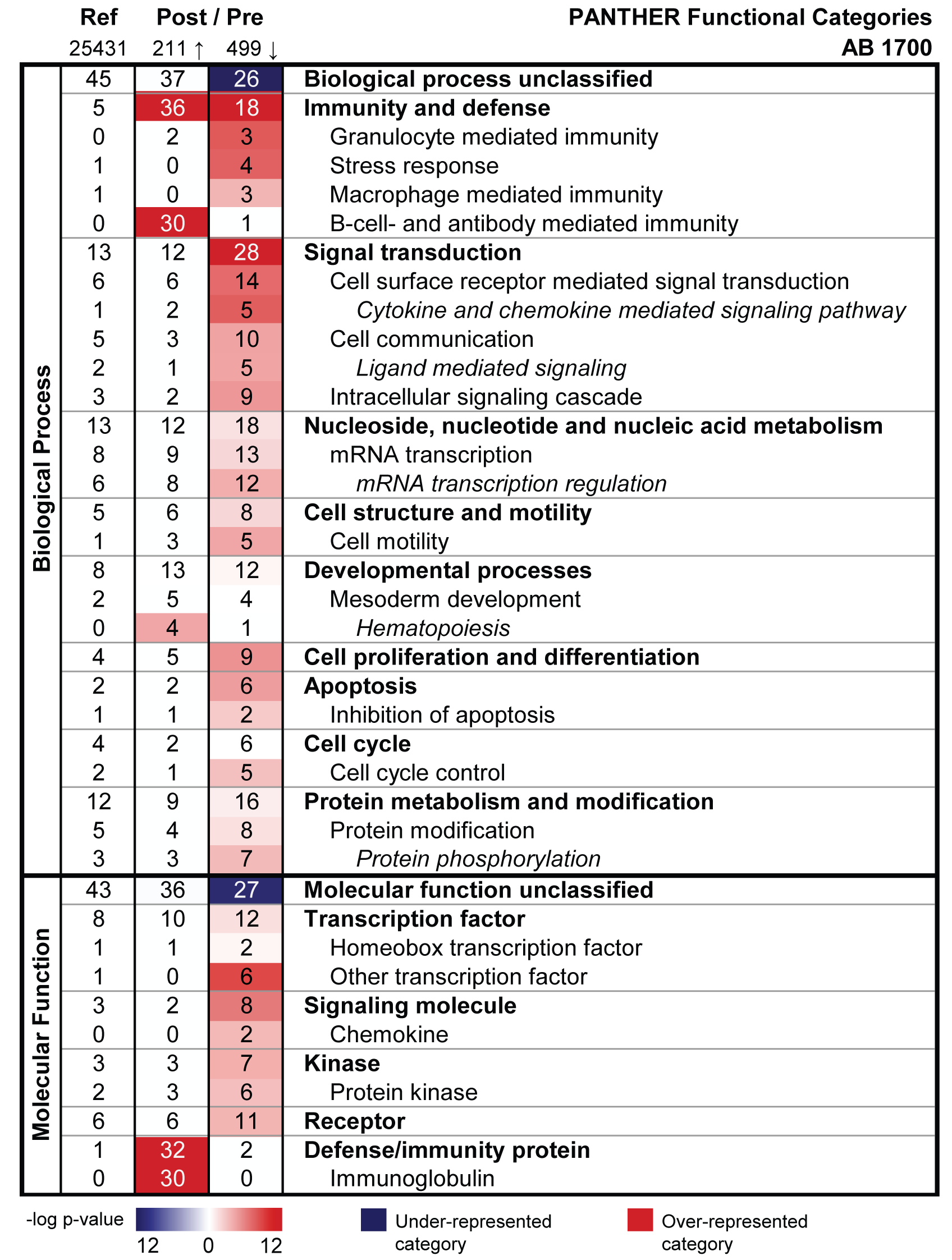

Supplement: Figure S1 — Functional categorization of differentially expressed genes in adipose tissue after fat loss (AB 1700, n = 9). PANTHER was used to search for over-represented functional categories among the most differentially expressed genes (q-value = 0, fold change at least 1.5). The color intensity displays the statistical significance (−log p-value) of over- and under-represented PANTHER functional categories. Red color signifies an over-representation of genes mapping to a certain term, blue color an under-representation and white a representation as expected based on the overall distribution on the array. A p-value<0.01 was used as inclusion criterion for categories, with Bonferroni correction for multiple testing. Numbers presented in the table indicate the percentage of genes within a gene set that map to the given category, e.g. 18% of the 499 down-regulated genes map to the biological process ‘Immunity and defense’. The first column states the overall distribution of a term among all human NCBI genes (25,431), e.g. 5% of the genes are expected to map to ‘Immunity and defense’, hence this category is significantly over-represented among the down-regulated genes. Of note, unlike the Illumina data, the data showed an up-regulation of genes involved in B-cell and antibody-mediated immunity (e.g. immunoglobulins). However, the majority of these genes had records that were discontinued in the Entrez Gene database or were listed as hypothetical proteins. Ref, reference (based on all human NCBI genes); Pre, pre-surgery biopsies; Post, post-surgery biopsies; Ctr, lean controls; Arrow up, up-regulated/more expressed genes; Arrow down, down-regulated/less expressed genes (e.g. arrow up in Ctr/Post signifies higher expression in Ctr). (0.48 MB TIF) [file pone.0011033.s010.tif]
